# Collagen denaturation in post-run Achilles tendons and Achilles tendinopathy: In vivo mechanophysiology and magnetic resonance imaging

**DOI:** 10.1126/sciadv.ado2015

**Published:** 2024-10-02

**Authors:** Yijie Fang, Dantian Zhu, Jingyue Wei, Lei Qian, Rongmao Qiu, Taoyu Jia, Kui Huang, Suwen Zhao, Jun Ouyang, Man Li, Shaolin Li, Yang Li

**Affiliations:** ^1^ Department of Radiology, Guangdong Provincial Engineering Research Center of Molecular Imaging, Guangdong-Hong Kong-Macao University Joint Laboratory of Interventional Medicine, the Fifth Affiliated Hospital, Sun Yat-sen University, Zhuhai, Guangdong 519000, China.; ^2^ Guangdong Provincial Engineering Research Center of Molecular Imaging, Biobank, Department of Information Technology and Data Center, the Fifth Affiliated Hospital, Sun Yat-sen University, Zhuhai, Guangdong 519000, China.; ^3^ Guangdong Provincial Key Laboratory of Digital Medicine and Biomechanics, Guangdong Engineering Research Center for Translation of Medical 3D Printing Application, National Virtual & Reality Experimental Education Center for Medical Morphology, National Experimental Education Demonstration Center for Basic Medical Sciences, National Key Discipline of Human Anatomy, School of Basic Medical Sciences, Southern Medical University, Guangzhou, Guangdong 510000, China.; ^4^ Department of Oral and Maxillofacial Surgery, The Affiliated Stomatological Hospital of Southwest Medical University, Luzhou, Sichuan 646000, China.

## Abstract

Achilles tendinopathy is often attributed to overuse, but its pathophysiology remains poorly understood. Disruption to the molecular structure of collagen is fundamental for the onset and progression of tendinopathy but has mostly been investigated in vitro. Here, we interrogated the in vivo molecular structure changes of collagen in rat Achilles tendons following treadmill running. Unexpectedly, the tendons’ collagen molecules were not mechanically unfolded by running but denatured through proteolysis during physiological post-run remodeling. We further revealed that running induces inflammatory gene expressions in Achilles tendons and that long-term running causes prolonged, elevated collagen degradation, leading to the accumulation of denatured collagen and tendinopathy development. For applications, we demonstrated magnetic resonance imaging of collagenase-induced Achilles tendon injury in vivo using a denatured collagen targeting contrast agent. Our findings may help close the knowledge gaps in the mechanobiology and pathogenesis of Achilles tendinopathy and initiate new strategies for its imaging-based diagnosis.

## Teaser

Achilles tendons’ collagen molecules are denatured in vivo by protease degradation during post-run tissue remodeling.

## INTRODUCTION

Achilles tendinopathy is an increasingly prevalent condition in the general population and substantially affects the careers of more than 20% of athletes (*1*, *2*). The Achilles tendon is the thickest tendon in the human body. It is responsible for transmitting the force generated by the foot’s plantar flexor muscles and maintaining stability and proper movement of the lower limb (*3*). The predominant component of the Achilles tendon is type I collagen, which exhibits a hierarchical fibrous structure (*4*). Disruption of the collagen fibers represents a notable pathological manifestation of Achilles tendinopathy and is closely associated with the progression of the condition (*1*, *5*, *6*). Although prolonged fatigue loading has been recognized as a crucial independent risk factor for Achilles tendinopathy, the underlying pathophysiology involved in the progressive damage to the structure of this collagenous tissue remains poorly understood (*1*, *7*).

In recent years, a collagen hybridizing peptide (CHP) has been developed to specifically bind to denatured collagen molecules with the unfolded triple-helical structure; it has enabled molecular detection of damage to collagen caused by overloading or cyclic loading of tendon tissue in situ (*5*, *8*). Using this collagen hybridization technique, investigations on tendon biomechanics have revealed the structure-property relationship of tendons with different anatomical functions from a molecular level (*9*, *10*) and indicated that denatured collagen molecules can accumulate during cyclic fatigue loadings before changes in local tissue mechanics (*11*–*14*). Nonetheless, these biomechanical studies have exclusively relied on in vitro and (rarely) ex vivo experiments (*5*, *9*–*15*), sometimes with damages occurring at the physical limits of the tissues (e.g., rupture) (*5*, *9*). Consequently, whether mechanical disruptions occur to the collagen molecules in vivo in tendons due to physical activities is still unknown.

The Achilles tendon is a living tissue that exhibits dynamic responses to stimuli (*6*, *7*, *16*). Current tendon mechanobiology research often relies on in vitro cell assays and ex vivo tests on living explants under mechanical loading (*15*, *17*–*23*). Meanwhile, most animal studies focus on tendon healing from acute injuries, such as a sharp transection (*24*–*31*). In contrast, in vivo investigations on the matrix and cellular responses to mechanical stimuli or overuse in healthy tendons are limited to date, most of which were carried out through exerting controlled fatigue loading on anesthetized animals (*32*–*36*). These studies have reported changes in the number and morphology of fibrocytes (*37*), as well as the expression of type III collagen and factors corresponding to oxidative stress (e.g., inducible nitric oxide synthase) in rat Achilles tendons after fatigue loading (*34*). Meanwhile, few clinical microdialysis studies in humans after physical exercises have detected elevated expressions and activities of matrix metalloproteinases (MMPs) and their inhibitors (*38*), as well as peptide markers of collagen synthesis and degradation in tissues surrounding the Achilles tendon (*39*, *40*).

These studies suggest that the collagenous tendon tissue undergoes biological remodeling during fatigue loading. Still strikingly little is known about the mechanisms by which healthy tendon accumulates damage (*6*, *41*). A series of topics concerning collagen structure and remodeling remains to be explored in vivo to better elucidate the pathophysiology of Achilles tendinopathy, such as the changes in the structure of collagen molecules in the Achilles tendon following exercises, the distinctive roles of mechanical loading and cellular responses in this process, and the impact of overuse on the collagenous architecture. Furthermore, diagnostic methods for Achilles tendinopathy are lacking unless the tissue morphology has already been irreversibly altered. For instance, conventional magnetic resonance imaging (MRI), the most common imaging modality for musculoskeletal soft tissues, does not visualize the Achilles tendon including its internal structures and lesions (*42*–*44*). Therefore, studying the subtle changes in the collagen structure of fatigued Achilles tendons in vivo may lead to much-needed new imaging biomarkers for detecting and assessing tendinopathy and injuries (*45*).

Here, utilizing the CHPs, we interrogated the in vivo changes in the micro- and molecular structure of collagen along with cellular responses in rat Achilles tendons following short- and long-term treadmill running. Our findings may help close the knowledge gaps in tendon mechanophysiology and the pathophysiology of Achilles tendinopathy. They may also lead to new MR contrast agents for noninvasive detection and imaging of Achilles tendinopathy.

## RESULTS

### In vivo collagen denaturation in the post-run Achilles tendons

**Fig. 1.**
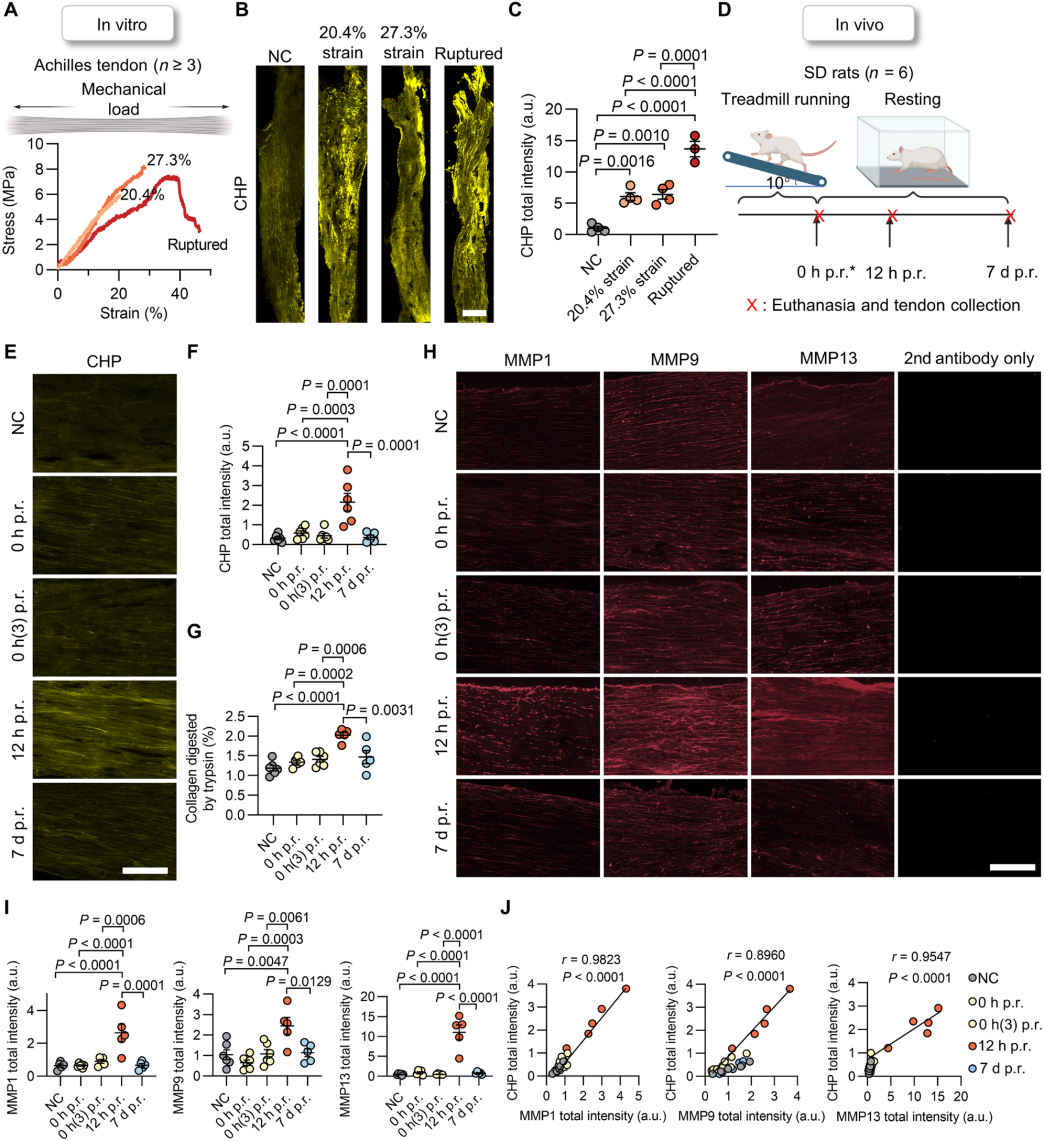
In vivo collagen denaturation in the post-run Achilles tendons. (**A**) Representative stress-strain curves of SD rats’ Achilles tendons following in vitro monotonical straining to 20.4 and 27.3% (~60 and 80% of the failure strain) or rupture. (**B** and **C**) Representative images (B) and quantified fluorescence (C) of the Cy5-CHP–stained whole-tendon cryosections from (A). (**D**) Design of the one-time treadmill study. After 1 hour of uphill running, rats were sacrificed with their Achilles tendons collected at 0 hours (immediately) p.r., 12 hours p.r., and 7 days p.r. (p.r., post-running). *0 hours (3) p.r.: run for 3 hours with tendons collected at 0 hours p.r.. NC: rats in the control group maintained normal activity without running until they were sacrificed simultaneously with the 12-hour p.r. group. (**E**) Representative Cy5-CHP staining of the Achilles tendon cryosections. (**F**) Quantified total Cy5-CHP fluorescence of the whole-section scans of the Achilles tendons (fig. S4). (**G**) Denatured collagen contents in the Achilles tendons by the trypsin-hydroxyproline assay. (**H** and **I**) Representative images (H) and quantitative analysis (I) of the whole-cryosection scans of the Achilles tendons immunostained against MMP1, MMP9, and MMP13 (fig. S4). The sections in the second antibody–only group were stained without primary antibodies (fig. S6). (**J**) Correlations between the histologic MMP and CHP signals. *r*: Pearson correlation coefficient. Each image represents results from at least three (B) or five rats per group [(E) and (H)]. Scale bars, 1 mm (B) and 150 μm [(E) and (H)]. Data were expressed by means ± standard error of the mean; statistical analysis: one-way analysis of variance (ANOVA) and Tukey [(C), (F), (G), and (I)]. Panels (A) and (D) were created with BioRender.com. a.u., arbitrary units.

We first confirmed the ability of CHP to recognize the collagen molecules denatured by mechanical loading in vitro in the Achilles tendon (Fig. 1, A to C). Full-size Achilles tendons of Sprague-Dawley (SD) rats were subjected to incremental strains up to complete failure (Fig. 1A) with a mechanical test system (fig. S1A). After loading and unloading, each tendon sample was cryosectioned longitudinally and stained with cyanine 5 (Cy5)–labeled CHP (Cy5-CHP) before being washed and fluorescently scanned (Fig. 1B). The measured Cy5-CHP fluorescence intensity increased remarkably with the applied high strain (Fig. 1B and figs. S2 and S3): the 20.4 and 27.3% strained groups (i.e., approximately 60 and 80% of the failure strain) exhibited intensities several folds greater than the unloaded control (Fig. 1C), confirming that monotonical loading to nonphysiological strains can denature collagen molecules in rat Achilles tendons.

In contrast, to find out whether physiological-level exercises can cause collagen denaturation in the Achilles tendon in vivo, SD rats were made to run uphill for 1 hour on a treadmill (Fig. 1D; fig. S1, B and C; tables S1 and S2; and Movie S1), and their Achilles tendons were collected immediately post-running (0 hour p.r.) or after 12 hours or 7 days of rest (12 hours p.r. and 7 days p.r., Fig. 1D); their left Achilles tendons were also cryosectioned longitudinally for Cy5-CHP staining and fluorescence scanning. Unexpectedly, the CHP fluorescence intensity of the tendons did not increase after running compared to the control rats at rest (Fig. 1, E and F, and fig. S4A); no higher-than-normal collagen denaturation was detected even with 3 hours of continuous running [0 hours (3) p.r.] and some rats’ paws swelling up (Fig. 1, E and F). To verify this, we collected the right Achilles tendons of these rats and treated them with trypsin, which selectively digests denatured collagen from the tissue, allowing us to determine the denatured collagen content in the tendons by measuring the cleaved collagen in the solutions by the hydroxyproline assay (*5*). This trypsin-hydroxyproline test also revealed no significant increase in collagen denaturation in the Achilles tendons with 1 or 3 hours of running (Fig. 1G), suggesting that even intense exercise may not cause the collagen molecules to unfold in the Achilles tendon in vivo.

Intriguingly, the Cy5-CHP staining and the trypsin-hydroxyproline test both indicated that the denatured collagen content in the Achilles tendons increased notably at 12 hours p.r. but decreased to nearly the baseline level after 7 days of rest. At 12 hours p.r., the CHP fluorescence intensity was fourfolds that of the control group (Fig. 1, E and F, and fig. S4; also see fig. S5 for negative results from our sequence-scrambled control peptide); correspondingly, an additional 0.6 to 0.8% of the tendons’ total collagen became denatured at 12 hours p.r. compared to the control and the 0-hour p.r. groups (Fig. 1G). Meanwhile, the collagen denaturation level for the 7-day p.r. group was not significantly different from the resting control or the 0-hour p.r. groups (Fig. 1, F and G). In seeking possible biological factors of collagen denaturation of the Achilles tendon during post-run resting, we carried out immunofluorescence staining of the tendon sections against MMPs (Fig. 1H and fig. S6). We found that the concentrations of MMP1, MMP9, and MMP13 in the Achilles tendon remained at the prerun level at 0 hours p.r. but rose considerably at 12 hours (especially MMP13) before decreasing to baseline at 7 days (Fig. 1, H and I, and fig. S4). The quantified fluorescence intensities from the MMP immunostainings were nearly proportional to those of the CHP stain in a linear fashion, indicating strong correlations (Fig. 1J). Meanwhile, quantitative real-time reverse transcription polymerase chain reaction (qRT-PCR) analysis showed that the gene transcription of *Mmp13* and *Mmp1* was also up-regulated in the Achilles tendons at 0 and 12 hours p.r., respectively (fig. S7), supporting our MMP immunofluorescence findings. Last, we further profiled collagen denaturation during the latter stage of recovery at 12 hours, 2 days, 4 days, and 7 days p.r. in a separate animal study (fig. S8A). The CHP intensity and immunofluorescence of the MMPs had all decreased to almost baseline levels around 4 days p.r. (fig. S8, B and C) while consistently maintaining a linear correlation during this phase (fig. S8D).

### Restoration of collagen fiber organization in rat Achilles tendons during post-run resting

**Fig. 2.**
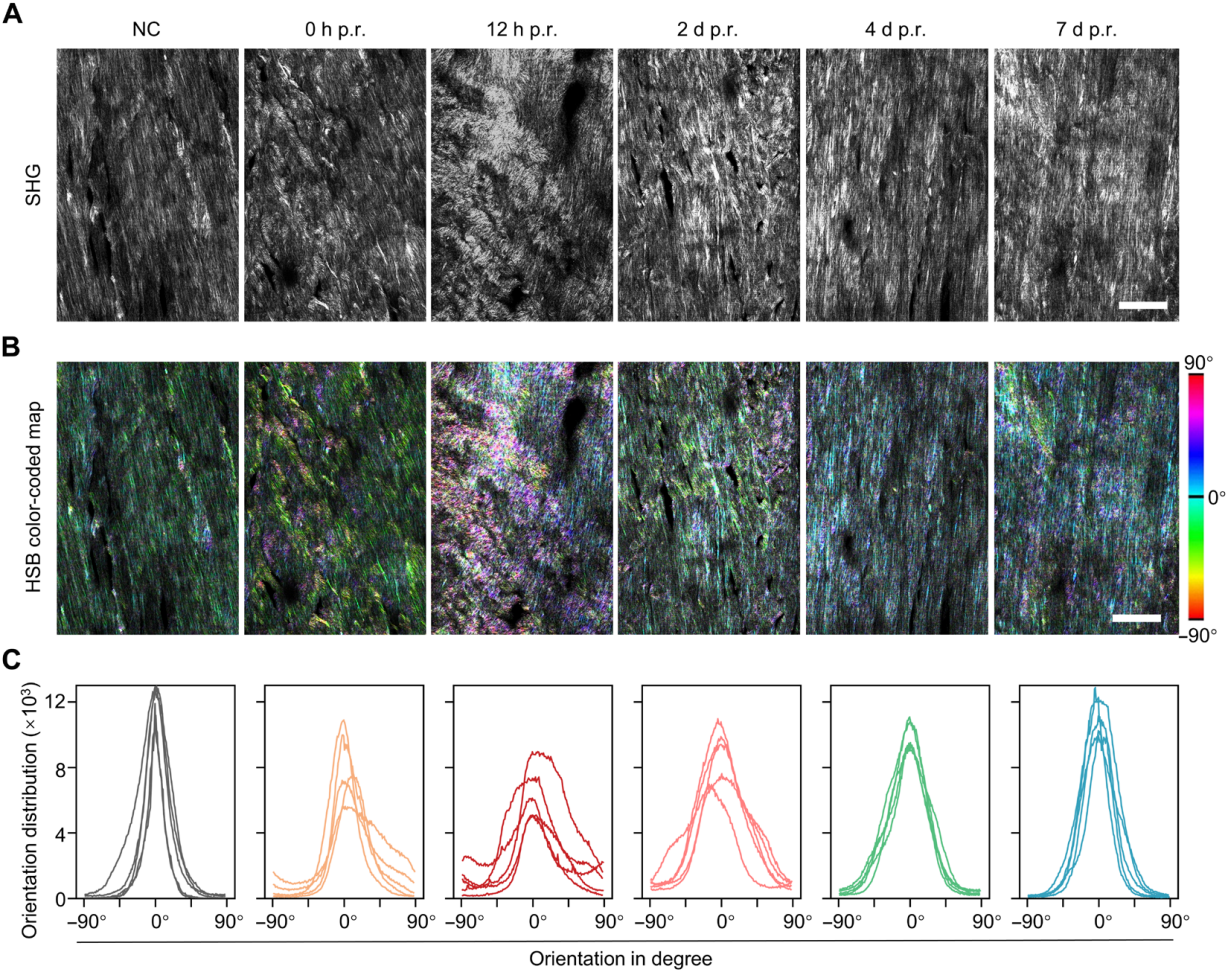
Collagen fiber disorganization in the post-run rat Achilles tendons. (**A** and **B**) Representative micrographs of the cryosections of the Achilles tendons from the one-time treadmill study acquired with SHG microscopy (A) and their HSB color-coded maps for visualization of fiber orientation (B) (Hue: local orientation; Saturation: coherency; Brightness: original image). (**C**) Distribution density plots counting the total number of pixels with a defined orientation (1-degree resolution) within each of the five microscopic views randomly acquired for each group. Additional images are in fig. S9. Scale bar, 100 μm.

We examined the collagen fiber alignment within the post-run rat Achilles tendons using second-harmonic generation (SHG) microscopy. These SHG images and their corresponding pixel orientation distribution plots showed that the originally well-aligned collagen fibers became disorganized and undulated immediately after running; this morphologic disorganization became less and less obvious from 2 to 7 days p.r. (Fig. 2, A and B, and fig. S9), with the fiber orientation becoming progressively more consistent (Fig. 2C).

### Morphological changes of Achilles tenocytes with elevated MMP and α-SMA expressions during post-run resting

**Fig. 3.**
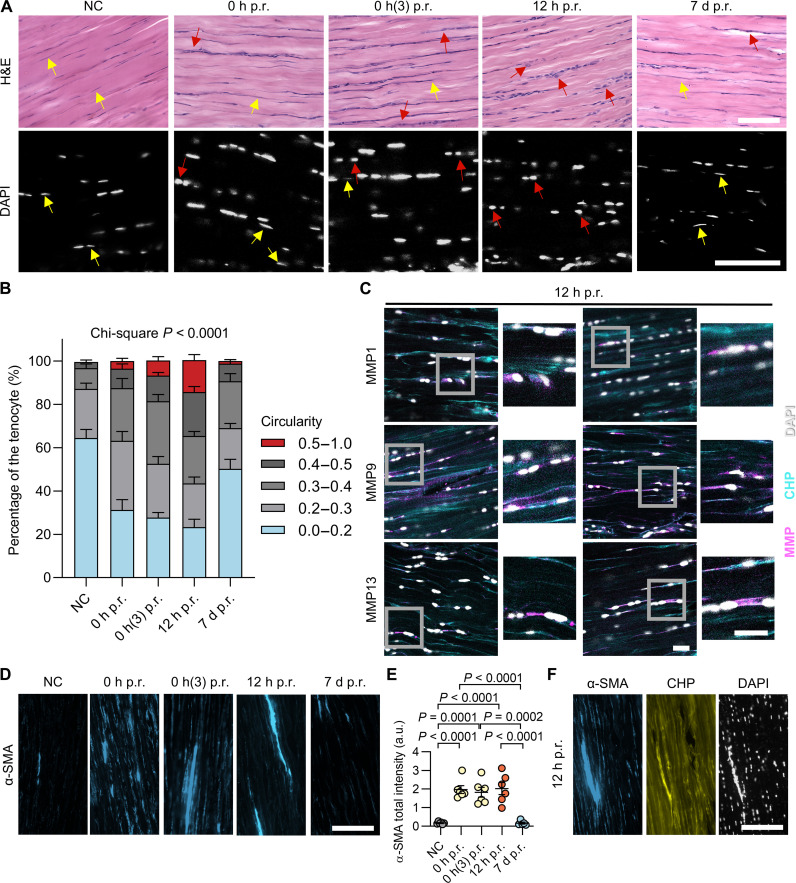
Achilles tenocytes undergo morphological changes and express MMPs and α-SMA during post-run resting. (**A**) Micrographs showing the H&E and DAPI staining of the Achilles tendon cryosections from the one-time treadmill running groups (yellow arrows: elongated nuclei, red arrows: round nuclei). (**B**) Percentage of the tenocytes with various nuclei circularities quantified from the confocal images of the DAPI staining of 12 Achilles tendon slides for each group (two fields of view per slide, *P* < 0.0001, chi-squared test). (**C**) Representative confocal scanning images of the Achilles tendon cryosections collected at 12 hours p.r., stained with DAPI, Cy5-CHP, and anti-MMP antibodies. (**D** and **E**) Immunostaining of α-SMA (D) and quantitative analysis (E) of the cryosections of the Achilles tendons from each group of the one-time treadmill experiment. Representative whole-section scans are shown in fig. S13. (**F**) Representative fluorescence images of the cryosections of the Achilles tendons from the 12-hour p.r. group, stained with DAPI, Cy5-CHP, and an anti–α-SMA antibody. The images represent similar results from at least five rats within each group [(A), (C), (D), and (F)]. Scale bars, 75 μm [(A), (D), and (F)] and 25 μm (C). Data were expressed as mean and standard error of the mean [(B) and (E)]; statistical analysis: one-way ANOVA and Tukey (E).

We carefully examined the histological and cellular features of the Achilles tendons at each time point. Through hematoxylin and eosin (H&E) stain and 4′,6-diamidino-2-phenylindole (DAPI) staining, we noted that the cell nuclei in Achilles tendons changed from the elongated morphology for the control group to a more circular shape beginning at 0 hours p.r.; this change became most evident at 12 hours p.r. (Fig. 3A and fig. S10). Immunofluorescence against tenomodulin (TNMD) (*46*) confirmed the tenocyte identity of these cells with rounded nuclei (fig. S11). According to quantitative image analysis, the percentage of rounded nuclei increased progressively in the order of the control group, the 0 hours p.r. group, and 12 hours p.r. group (chi-square test, χ^2^ < 0.0001) (Fig. 3B). After 2 to 7 days of rest, most nuclei gradually restored the elongated shape similar to the control group (Fig. 3A and fig. S12). Moreover, the merged fluorescence images revealed that the emerging MMP1, MMP9, and MMP13 peaked at 12 hours p.r. (figs. S10 and S12) and seemed to be mostly located adjacent to the cells with rounded nuclei (Fig. 3C). Furthermore, as a marker of activated fibroblasts, the tenocytes’ α-smooth muscle actin (α-SMA) expression increased markedly, starting from 0 hours p.r., remaining elevated at 12 hours p.r., and becoming barely detectable by day 7, as revealed by our immunofluorescence stain (Fig. 3, D and E, and fig. S13). We also noted elevated CHP fluorescence intensity in close proximity to the activated tenocytes with a high α-SMA expression at 12 hours p.r. (Fig. 3F and fig. S14).

### Altered gene expressions in the post-run Achilles tendons

**Fig. 4.**
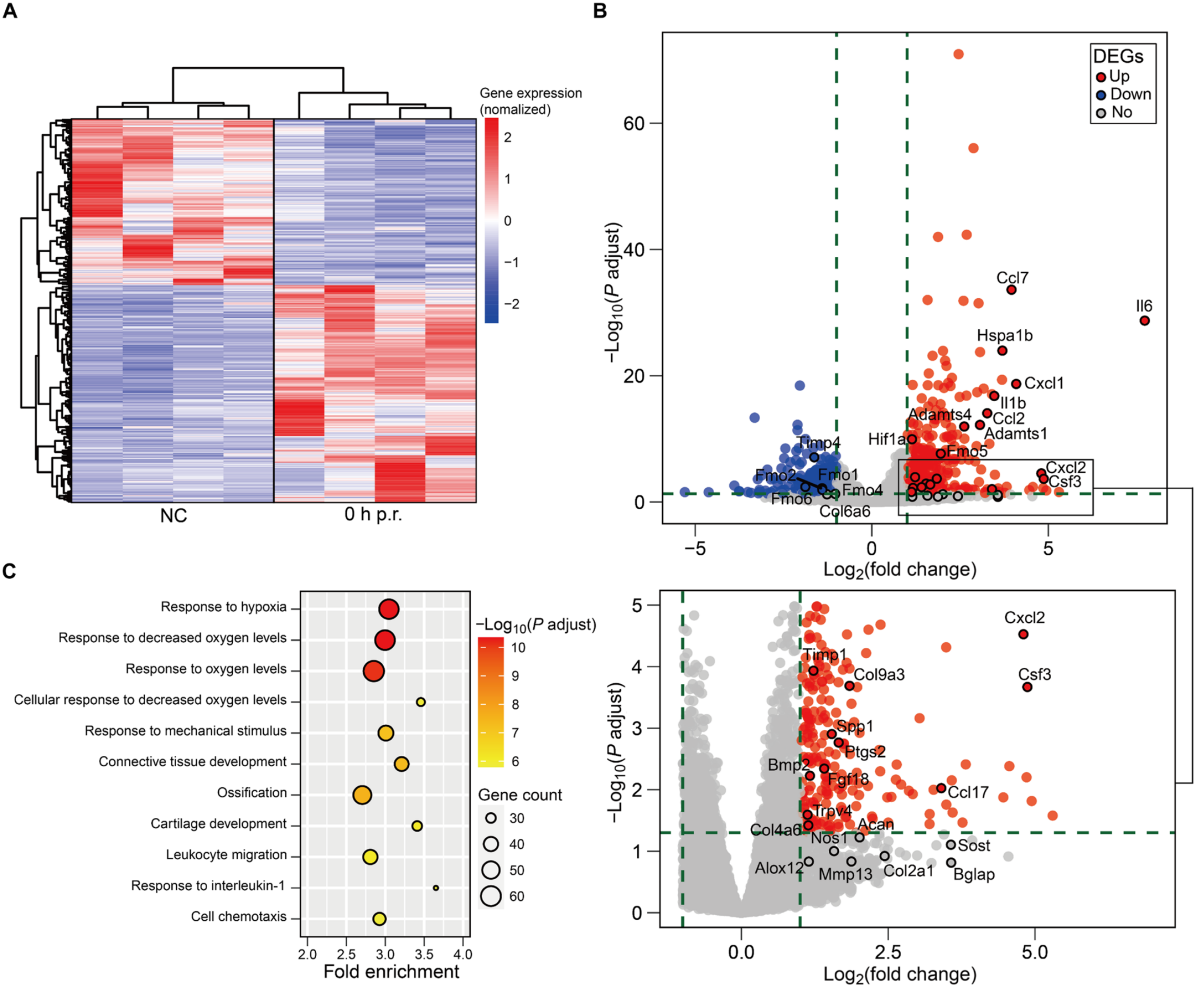
RNA-seq analysis of the tendons from the NC and 0-hour p.r. rats. (**A**) Heatmap of all differentiated expressed genes (DEGs) for the Achilles tendons in the NC and 0-hour p.r. rats. (**B**) Volcano plot of the differentially expressed genes (0 hour p.r. versus NC; fold change ≥ 2; adjust *P* value < 0.05). Significantly up-regulated genes are represented in red. Significantly down-regulated genes are in blue. Nonsignificant genes are in gray. Green vertical dotted lines highlight log fold changes of −1 and 1, whereas the green horizontal dotted line represents −log_10_(adjust *P* value) of 1.30. (**C**) Gene Ontology enrichment analysis revealed that 1 hour of running resulted in the rapid enrichment of key biological processes in the rat Achilles tendons, such as response to hypoxia and mechanical stimulus, inflammation, connective tissue development, and ossification.

To profile the in vivo biological responses of the Achilles tendons to running, we conducted RNA sequencing (RNA-seq) using the Achilles tendon tissues collected from the rats at rest (naïve control, NC) or immediately following 1-hour treadmill running (0 hours p.r.). Compared to the NC group, we found 358 up-regulated and 281 down-regulated protein-coding genes in the post-run Achilles tendons (fold change ≥ 2; adjust *P* value < 0.05) (Fig. 4, A and B). In terms of extracellular matrix (ECM) remodeling, we noted that the *Mmp13* gene expression was moderately up-regulated following running, although this change did not appear statistically significant within the whole transcriptome (fold change = 3.66, *P* value = 0.025, adjust *P* value = 0.15; Fig. 4B). Besides, the endogenous MMP-inhibitor *Timp1* was also up-regulated after running (Fig. 4B). Moreover, aggrecan gene *Acan* and proteoglycan-degrading aggrecanases *Adamts1* and *Adamts4* were up-regulated (Fig. 4B), suggesting proteoglycan turnover (Fig. 4B). In addition, *Il6*, a cytokine that can induce MMP production and matrix degradation (*47*, *48*), was notably up-regulated in the 0-hour p.r. tendons (fold change = 212.56, adjust *P* value = 1.86 × 10^−29^) (Fig. 4B). Besides ECM remodeling, treadmill running resulted in up-regulation of genes associated with inflammatory responses, such as interleukins (*Il6* and *Il1b*), chemokines (*Cxcl1*, *Cxcl2*, *Ccl2*, *Ccl7*, and *Ccl17*), and factors of the nitric oxide (NO) and prostaglandins (PGs) synthesis pathway (*Nos1*, *Ptgs2*, and *Alox12*) (Fig. 4B). Our positive immunofluorescence against IL-6 within the tendon cryosections further supported these RNA-seq findings (fig. S15). Gene Ontology (GO) enrichment analysis indicated that a series of biological processes were enriched in the tendons after running, including responses to hypoxia and mechanical stimulus, inflammatory cell migration and chemotaxis, connective tissue development, and ossification (Fig. 4C and table S3).

We further conducted RNA-seq using the 12-hour p.r. Achilles tendons. Compared to the NC group, we only found 51 up-regulated and 42 down-regulated protein-coding genes, suggesting that gene transcription was returning to normal in the rats’ Achilles tendons after 12 hours (fig. S16A versus Fig. 4B). Meanwhile, biological processes, such as responses to hypoxia or mechanical stimulus were no longer significantly enriched (fig. S16B and table S4). Overall, our data reflected a range of rapidly changing mechanobiological responses in the post-run Achilles tendon (Fig. 4 and fig. S16).

### Accumulation of collagen denaturation in Achilles tendons during long-term running

**Fig. 5.**
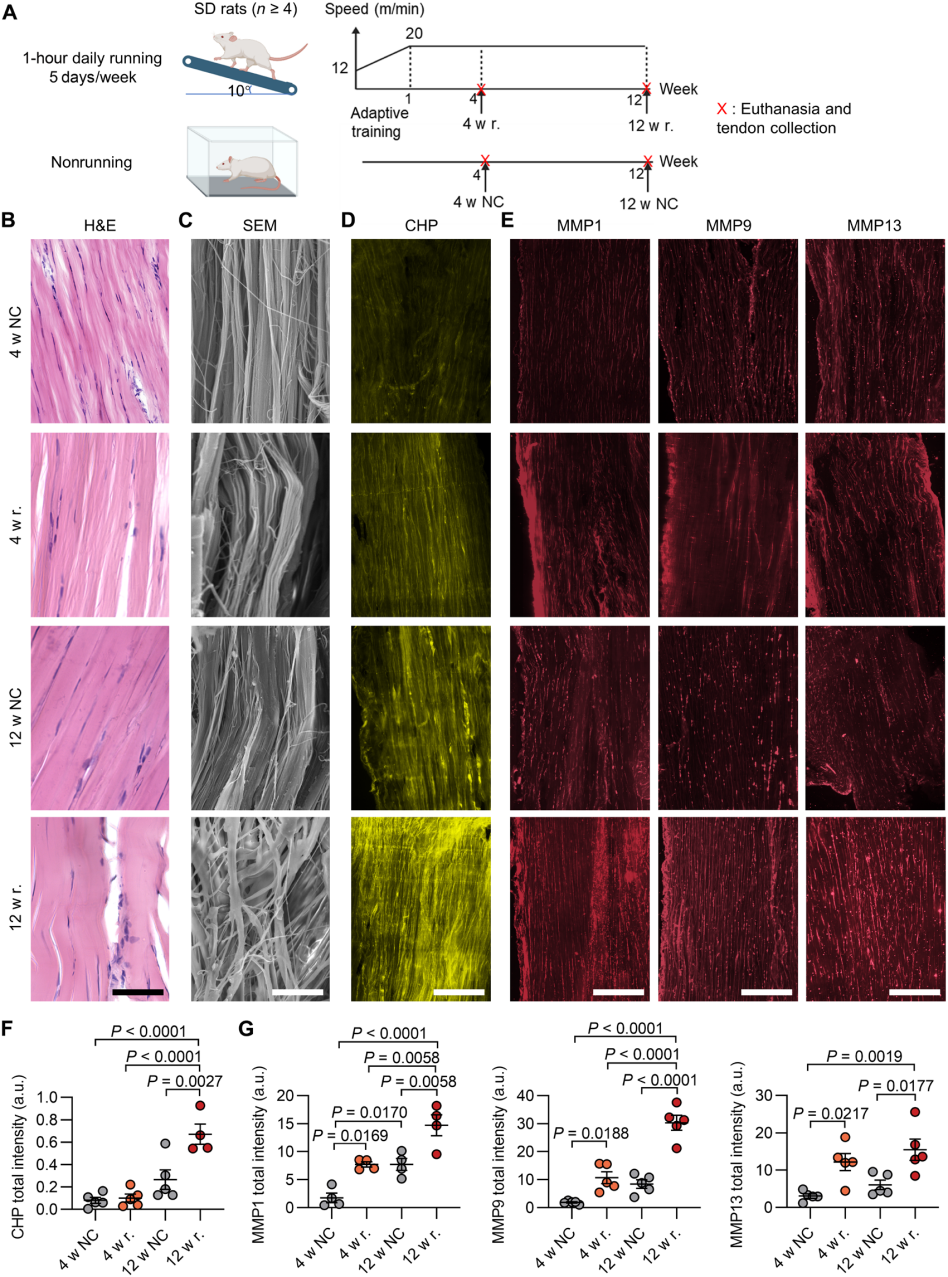
Accumulation of collagen denaturation in the rat Achilles tendons during long-term running. (**A**) Schematic of the long-term treadmill experiment. After running for 4 or 12 weeks, the rats from the running groups (4 w r. and 12 w r.) were euthanized, and the Achilles tendons were collected. Rats in the control group maintained normal activity in cages throughout the study (4 w NC and 12 w NC). To minimize the immediate effects of running, all rats were euthanized at the same time 24 hours after the running group finished the last 1-hour running session. (**B** to **E**) Micrographs of the H&E staining (B), SEM (C), Cy5-CHP staining (D), and the MMP1, MMP9, and MMP13 immunofluorescence (E) of the cryosections of the Achilles tendons obtained from each group of the long-term treadmill experiment. Representative whole-section scans are shown in fig. S17. (**F** and **G**) Quantified total Cy5-CHP (F) as well as MMP1, MMP9, and MMP13 (G) fluorescence intensities from the whole-section scans of the rats’ Achilles tendons collected from the control and long-term running groups. The images represent similar results from at least four rats within each group [(B) to (E)]. Scale bars, 75 μm [(B), (D), and (E)] and 8 μm (C). Data were expressed by mean ± standard error of the mean; statistical analysis: one-way ANOVA and Tukey [(F) and (G)]. Panel (A) was created with BioRender.com.

To understand the effect of prolonged fatigue on collagen denaturation in the Achilles tendon, we conducted a long-term running experiment, in which SD rats ran for 1 hour 5 days per week for 4 or 12 weeks (4 w r. and 12 w r.; Fig. 5A and table S5) (*37*). Afterward, we characterized the collagen structures at different scales and the MMP expressions of the Achilles tendons. Histologically, unlike the Achilles tendons from the nonrunning controls, whose collagen fibers were straightly aligned with narrow-shaped tenocytes in between, the fibers of the tendons from the 4- and 12-week running groups had an undulated morphology populated with rounded cell nuclei (Fig. 5B). Scanning electron microscope (SEM) imaging revealed that the collagen fibers of the tendons from the 12-week running group were disorganized, whereas the disruption to the fiber structural integrity was more recognizable for the 12-week running group (Fig. 5C).

The variation in CHP fluorescence intensity from the cryosection staining was barely discernable between individual tendons from the running and resting groups at week 4 (Fig. 5, D and F). However, the difference in CHP fluorescence became substantial between the running and resting groups at week 12 (Fig. 5, D and F, and fig. S17A). The average total CHP fluorescence intensity of the tendon sections from the 12-week running group was over sixfold that of the 4-week running group (Fig. 5, D and F). Meanwhile, the sequence-scrambled control peptide showed no binding to any slides, confirming that the CHP binding is specifically due to the triple-helix hybridization (fig. S5B). In addition, the expressions of MMP1, MMP9, and MMP13 were elevated after 4 weeks of running, while the expression of MMP1 and MMP9 continued to rise after 12 weeks of running (Fig. 5, E and G, and fig. S17B). These results suggested that extensive overuse over a prolonged period can result in elevated expression of collagen degrading factors in Achilles tendons, leading to constant remodeling with the accumulation of denatured collagen molecules within the disrupted matrix fibers.

### Probing collagen denaturation in vivo in a mouse model of collagenase-induced Achilles tendon injury

**Fig. 6.**
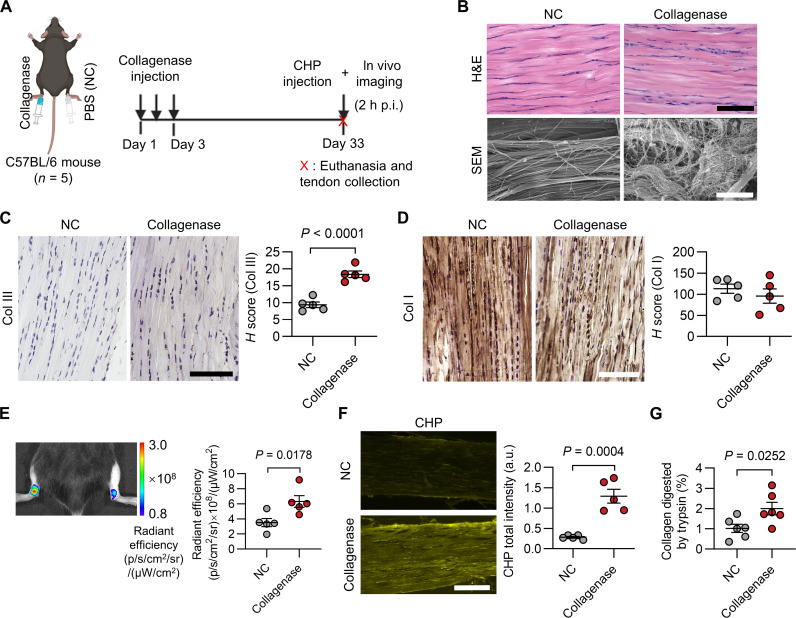
Probing collagen denaturation in the mouse model of collagenase-induced Achilles tendon injury through in vivo triple-helix hybridization. (**A**) Study timeline and schematic of the collagenase-induced Achilles tendon injury in the mice’s left ankles. (p.i.: post-injection). (**B**) H&E histology and SEM images of the model mice’s normal and diseased Achilles tendons. (**C** and **D**) Representative images and quantitative analysis of the cryosections of the model mice’s normal and diseased Achilles tendons, immunostained against type III (C) and I collagens (D). (**E**) In vivo fluorescence images and quantitative analysis of the collagenase-injured mouse model 2 hours after a subcutaneous and peritendinous injection of 0.04 nmol of Cy5-CHP to each ankle. (**F**) Fluorescence images and quantitative analysis of the Achilles tendon cryosections collected from the mice subcutaneously injected with Cy5-CHP in vivo. (**G**) Denatured collagen contents of both Achilles tendons from the mouse model mice measured by the trypsin-hydroxyproline test. The images are representative of similar results from five mice within each group [(B) to (F)]. Scale bars, 75 μm [(B): H&E, (C) and (D)], 8 μm [(B: SEM)], and 250 μm (F). Data were expressed by means ± standard error of the mean; statistical analysis: *t* test [(C) to (G)]. Panel (A) was created with BioRender.com.

To in vivo detect collagen denaturation in animal models that mimic the pathology of tendinopathy, we produced a mouse model with an injured Achilles tendon through three daily subcutaneous injections of collagenase to the left ankles of a group of C57BL/6 mice (Fig. 6A) (*49*); meanwhile, phosphate-buffered saline (PBS) was injected into their right ankles for comparison. After 30 days, the collagenase-treated Achilles tendons displayed clear histopathological characteristics of tendinopathy, such as clusters of round cell nuclei (Fig. 6B), disorganized collagen fibers with randomly aligned thin fibers (Fig. 6B), and minor changes in collagen composition (Fig. 6, C and D) (*50*). We injected an equal amount of Cy5-CHP subcutaneously into the peritendinous tissues of the left and right Achilles tendons of these mice in vivo. The fluorescence intensity from the Achilles tendon area of the collagenase-injected ankles was about twice that of the control side 2 hours post-CHP injection (Fig. 6E and also see fig. S18 for negative results from our sequence-scrambled control peptide). Also, the in situ fluorescence from the cryosections of the tendons dissected from the in vivo experiments showed strong CHP binding to the lesion throughout the depth of the whole tendon (Fig. 6F). Moreover, the trypsin-hydroxyproline test showed that the content of the trypsin-digestible denatured collagen in the collagenase-injected side was approximately twice that of the control side (Fig. 6G). Similarly, in a rat model of collagenase-induced Achilles tendon injury (*51*), we confirmed that the in vivo uptake of CHP in the collagenase-injected Achilles tendons was considerably greater than normal (fig. S19 and movie S2). Together, these findings demonstrated that CHP can successfully target the Achilles tendon’s denatured collagen in vivo, which may be a key molecular signature of the tendon matrix in the Achilles tendinopathy mimicking animal models.

### In vivo MRI of the collagenase-injured Achilles tendon

**Fig. 7.**
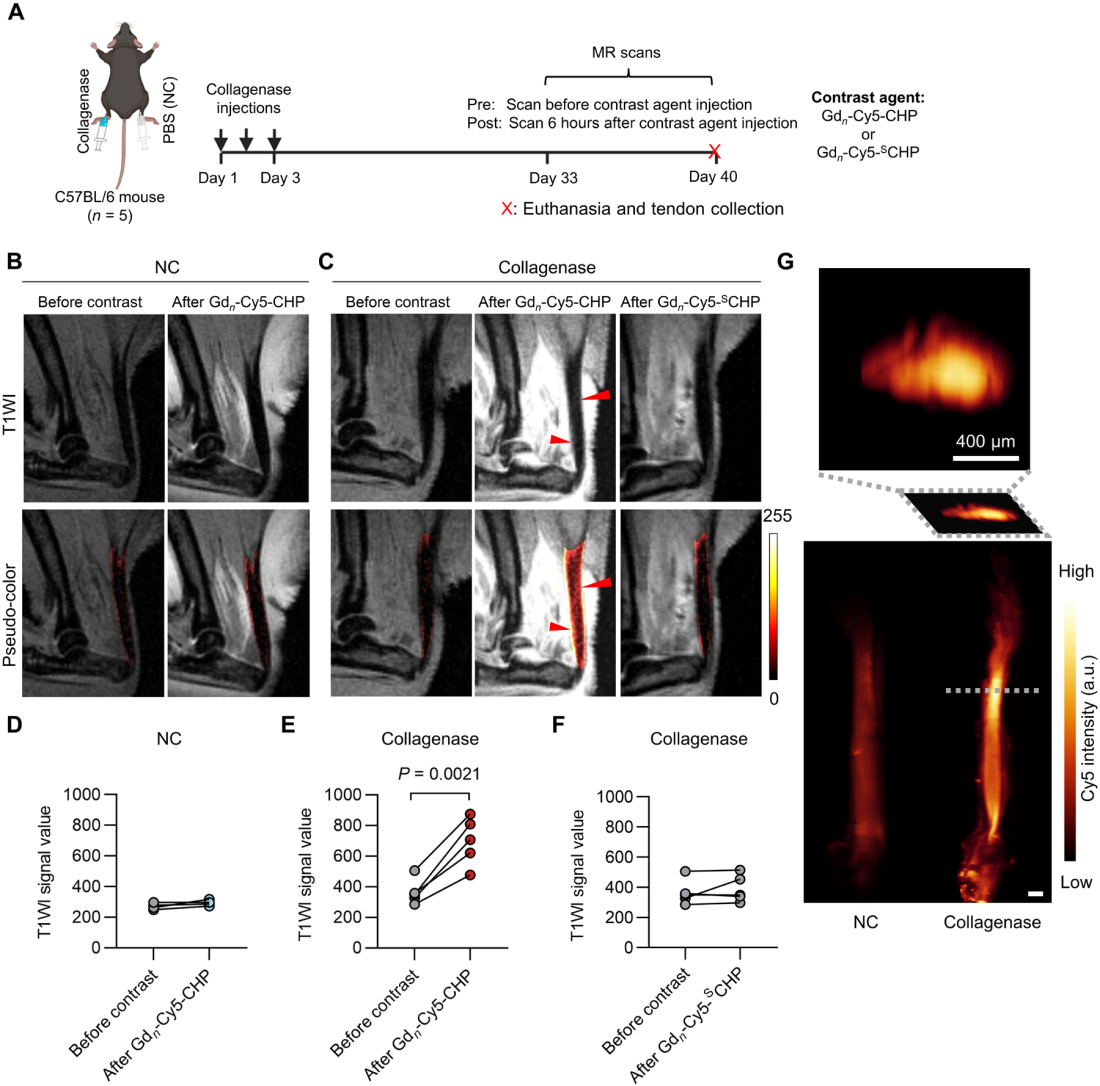
In vivo MR imaging of collagenase-induced Achilles tendon injury using Gd_*n*_-Cy5-CHP. (**A**) Study timeline, schematic of the mouse model, and the MR scan process. (**B** and **C**) Representative in vivo T1WI images (with and without pseudo-coloring within the Achilles tendons) of the normal (B) and collagenase-injured (C) ankles from the same mouse imaged on a 9.4-T MR scanner. The mice were live scanned before and 6 hours after peritendinous injection of 50 nmol of Gd_*n*_-Cy5-CHP or Gd_*n*_-Cy5-^S^CHP. The images were representative of results from five mice. (Red arrowheads: regions with notable enhancement). (**D**) Quantitative analysis of the in vivo T1W signals of the normal Achilles tendon before and after injection of Gd_*n*_-Cy5-CHP. (**E** and **F**) Quantitative analysis of the in vivo T1W signals from the Achilles tendons with collagenase-induced injury before and after injection of Gd_*n*_-Cy5-CHP (E) or Gd_*n*_-Cy5-^S^CHP (F). (**G**) Light-sheet fluorescence microscopy images of the Gd_*n*_-Cy5-CHP bound Achilles tendons collected from the model mice following in vivo MRI (top: cross-section view, bottom: 3D view). Differences in the T1W signal were compared using the paired *t* tests. Scale bars, 400 μm (G). Panel (A) was created with BioRender.com.

MR is the predominant clinical imaging tool for diagnosing diseases and injuries in the musculoskeletal system. However, it usually fails to detect Achilles tendinopathy early due to the lack of signals from the tendons (*42*–*44*). Toward the goal of visualizing Achilles tendinopathy by MRI, we synthesized a fluorescent MR contrast agent, namely, Gd*_n_*-Cy5-CHP, and its sequence-scrambled control probe Gd_*n*_-Cy5-^S^CHP, both with eight 1,4,7,10-tetraazacyclododecane-1,4,7,10-tetraacetic acid (DOTA) moieties for gadolinium (Gd) labeling (fig. S20A). Our final Gd_*n*_-Cy5-CHP product exhibited a relaxivity value nearly eightfold that of the clinical MR contrast agent Magnevist [gadopentetic acid (Gd-DTPA), T1 relaxivity per mM probe: 35.22 mM^−1^ s^−1^ versus 4.17 mM^−1^ s^−1^; fig. S20B]. In vitro MR imaging of heat-denatured Achilles tendons showed strong signals for the Gd_*n*_-Cy5-CHP–treated samples but not the ones incubated with Gd_*n*_-Cy5-^S^CHP, confirming the triple-helix–based specific binding of Gd_*n*_-Cy5-CHP to denatured collagen (fig. S20C). Next, we tested the probes in our mouse models of the collagenase-injured Achilles tendon by MRI (Fig. 7A). Gd_*n*_-Cy5-CHP was subcutaneously injected into the peri-Achilles tendon areas of each mouse’s two ankles for T1-weighted scanning. Before probe injection, both ankles of each mouse were prescanned with T2WI and T1WI. As expected, conventional sagittal scans with a T2W sequence showed neither enhanced signals nor marked thickening of the collagenase-injected Achilles tendons, failing to indicate the lesion (fig. S21A). Similarly, the T1WI prescans of the normal and collagenase-injected ankles showed no difference in signal (Fig. 7, B and C). In contrast, 6-hour post-probe injection, compared to the prescans, enhanced and heterogeneous signals could be observed in the diseased but not the normal Achilles tendons (Fig. 7, B and C). Meanwhile, the T1WI signals in the Achilles tendons from the collagenase-injected ankles were barely enhanced from the precontrast with Gd_*n*_-Cy5-^S^CHP (Fig. 7C, pseudo-color), suggesting that the enhanced signals in the Gd_*n*_-Cy5-CHP images were mostly due to the triple-helix hybridization of CHP with the denatured collagen in the diseased tendons. Quantitatively, the T1WI signals averaged from the regions of interest within the Achilles tendons (illustrated in fig. S21, B and C) were almost all doubled for each diseased tendon after Gd_*n*_-Cy5-CHP injection (Fig. 7E), whereas the T1WI signals remained unchanged for the normal Achilles tendons after Gd_*n*_-Cy5-CHP injection (Fig. 7D) and the diseased tendons after Gd_*n*_-Cy5-^S^CHP injection (Fig. 7F). Last, in addition to conventional histopathology (fig. S21D), we harvested these tendons following the in vivo MR scans and performed three-dimensional (3D) light sheet fluorescence microscopy imaging (Fig. 7G and movie S3), which showed strong but heterogeneously distributed Cy5 fluorescence intensity almost comparable to the T1WI signals from their individual in vivo MR images (Fig. 7C, pseudo-color). Together, we showcased the visualization of the collagenase-injured Achilles tendon in vivo via MRI by using a CHP-based MR probe targeting the denatured collagen.

## DISCUSSION

Using the collagen-hybridizing techniques, which enabled direct interrogation of the molecular structure of collagen (*5*), extensive in vitro studies have shown that mechanical loading can denature collagen molecules (*5*, *9*–*12*, *14*, *52*). These studies provided great insights into the structure-property relationship of tendons at the molecular level. A fraction of them demonstrated that cyclic loading of tendons of low tensile strength and modulus (e.g., mouse Achilles tendon) in vitro produces collagen fiber disorganization but minimal molecular denaturation (*9*, *10*, *53*). Gains *et al.* (*32*) detected denatured collagen molecules in the Achilles tendons of anesthetized rats overloaded in vivo (10,000 cycles at 3 Hz) but did not examine the underlying factors. Here, we ensured the rats ran continuously at a roughly steady speed on the treadmill (*37*, *54*), but no higher-than-normal collagen denaturation was detected in the Achilles tendons even after 3 hours of running (approximating 21,600 loading cycles at 2 Hz; Fig. 1). Our results provided strong support for the in vitro findings of Pedaprolu *et al.*(*53*), which suggested that tendinopathy may not be initiated by the mechanically induced collagen denaturation.

Our study suggests that the in vivo collagen denaturation of the post-run Achilles tendon is intricately involved in biological remodeling, probably as a mechanobiological mechanism to repair the physical microdamage to the tendon matrix (Figs. 1 to 3). Consistent with the previous fatigue loading findings from anesthetized rats’ patellar tendons in vivo (*33*, *35*, *36*) and mice’s Achilles tendons in vitro (*53*), we found that altered microstructures of the collagen fibers in exercised rat Achilles tendons immediately after running (Fig. 2 and fig. S9). Following 12 hours of rest, collagen denaturation became readily detectable in the tendon specimens (Fig. 1). Yet, this level of collagen denaturation is an order of magnitude lower than the ones found in the samples stretched to denature in vitro (fig. S3), also suggesting that the in vivo collagen denaturation is different from the unfolding caused by in vitro monotonical loading to failure. Collagen denaturation was noted in our in vivo study along with a series of cellular responses to mechanical stimuli, such as up-regulation of α-SMA, the rounded cell nuclei, and the expression of several MMPs (Figs. 1 to 3), which had been studied extensively in vitro and ex vivo (*15*, *17*–*23*). Most notably, the variation in MMP concentrations synchronizes with the degree of collagen denaturation throughout the recovery stages (Fig. 1 and figs. S4 and S8), where the CHP intensity is consistently proportional to the MMP immunofluorescence (Fig. 1J and fig. S8D). These results strongly suggested that collagen denaturation in a post-exercise Achilles tendon depends on protease-mediated collagen degradation and remodeling. Furthermore, after 4 to 7 days of rest, with the denatured collagen content decreasing to the minimal (Fig. 1, E and F, and fig. S8), the organized microstructure of collagen fibers was gradually restored to the morphology of exercise states (Fig. 2 and fig. S9). We speculate that mechanical loading during the exercise causes microdestruction to and/or relaxation of the collagen fibers (*32*, *33*, *35*, *36*, *53*), within which the load-stimulated tenocytes begin to degrade collagen molecules through factors such as MMPs, to remodel the collagen fibers and restore the tissue’s intact structure (Figs. 1 to 3) (*18*–*22*, *26*, *29*, *32*, *35*, *36*). It can thus be implied that even if the exercise is moderate and there is no mechanical unfolding of the collagen triple-helix, the collagen molecules may still be degraded and denatured during post-exercise repair and remodeling. Therefore, the denatured collagen in the Achilles tendon may be a common metabolic product of the tissue mechanobiology following exercises rather than a direct physical product of the mechanics (*9*, *53*). Meanwhile, we acknowledge the need for further investigation to demonstrate the causal effect of MMPs on collagen denaturation through MMP inhibition and conditional knockout strategies. These studies can help identify the key matrix proteases (possibly beyond what we detected here) that play a dominating role during post-run collagen remodeling in the Achilles tendon.

Our RNA-seq analysis revealed several aspects of Achilles tendon mechanobiology resulting from running. First, we found that the genes involved in cell responses to mechanical stimuli were rapidly up-regulated after running (Fig. 4C and table S3). For example, the *Trpv4* gene, a stretch-activated ion channel (Fig. 4B) (*55*), was up-regulated among other protein sensors and ion channels that may be important for tenocyte mechanotransduction in mechanically stimulated tissues for detecting, processing, and responding to the load signals. Second, genes related to reactive oxygen species production (*Fmo1*, *Fmo2*, *Fmo4*, and *Fmo6*) were down-regulated after running (Fig. 4B). This is consistent with previous reports by Eliasson *et al*. (*29*) on loaded, severed rat Achilles tendons, showing that genes related to oxidative stress were down-regulated by loading. Third, we noted that genes associated with cartilage development and ossification were also up-regulated (*Bmp2*, *Bglap*, *Sost*, and *Grem1*; Fig. 4B). This is in line with previous findings from a rat model of supraspinatus tendon overuse showing increased expressions of cartilage markers (*56*, *57*). Fourth, we found that the expression of *Mmp1*, *Mmp13*, *Timp1*, and several other genes (e.g., *Adamts1* and *Adamts4*) associated with ECM remodeling (Fig. 4B and fig. S16) started to increase immediately following running. Notably, the up-regulation of several matrix proteases and inhibitors was implicated in human tendinopathy including *Mmp2*, *Mmp9*, *Mmp14*, *Mmp13*, *Mmp19*, and *Timp1* (*58*). Together, our transcriptome data profile the tendons’ overall cellular reactions to continuous running, highlighting a primarily pro-inflammatory and remodeling response (Fig. 4C and table S3) that weakens after 12 hours (fig. S16). These findings put our collagen denaturing and cellular remodeling results (Figs. 1 to 3) into perspective and show implications for fatigue-induced Achilles tendinopathy. Our RNA-seq data and analyses of healthy tendons can serve as a useful reference for future investigations in tendon mechanobiology and sports medicine.

Fatigue loading and overuse are considered the major causes of Achilles tendinopathy (*1*, *12*), yet we are still far from understanding the actual mechanisms by which healthy tendon accumulates damage (*6*). In this work, we found that during 12 weeks of running, denatured collagen molecules accumulated in the rats’ Achilles tendons concomitantly with increasing MMP concentrations (Fig. 5). Eventually, the tissue manifested local pathological characteristics of tendinopathy (Fig. 5, B and C) (*37*). Our one-time running test suggested that the post-exercise collagen remodeling in tendons requires an appropriate (if not lengthy) recovery period to restore the organized collagen fiber morphology probably through MMP-mediated degradation and remodeling of collagen (Figs. 1 to 3); our long-term running experiment suggested that without enough rest, the repeated, incomplete remodeling can result in the accumulation of not only unrepaired, disorganized collagen fibers (Fig. 5, B and C) but also denatured collagen molecules probably from the ongoing collagenolytic process (Fig. 5). The buildup of disrupted fibers and denatured collagen molecules could both compromise the mechanical property of the tissue, making it prone to injury and degeneration (*54*, *59*). Our study examined the dynamics of collagen denaturation induced by active physical exercises in vivo, providing direct molecular evidence supporting the notion that chronic fatigue loading is responsible for the pathophysiology of Achilles tendinopathy.

Visualizing collagen structural disruption is a straightforward and effective approach for interrogating pathological changes in the Achilles tendons. New imaging biomarkers of tendon injury and disease are critically needed to better understand the clinical implications of altered tendon mechanobiology (*45*). Conventional analyses primarily focus on measuring proteases such as MMPs and a disintegrin and metalloproteinase with thrombospondin motifs (ADAMTSs), which cannot directly describe the structural damage within the tendon matrix (*38*, *60*, *61*). Moreover, the measurement itself usually requires destroying the tissue samples, which limits the clinical applications. Meanwhile, despite the versatile applications of MRI in musculoskeletal imaging (*62*), the Achilles tendon remains nearly invisible on conventional MRI: it has a very short echo time, which only provides extremely low signals (*63*). To visualize collagen damage in MRI, we developed a CHP-functionalized multilabeled contrast agent Gd_*n*_-Cy5-CHP and demonstrated in vivo MR imaging of Achilles tendon injury for the first time in a common collagenase-induced mouse model (Fig. 7) (*49*), showing superior results to conventional MRI and proving the concept that the damaged collagen can serve as a molecular marker for diagnostic tendon imaging. In the future, collagen probes with higher sensitivity and tissue-penetrating capacity need to be developed for early diagnosis and accurate monitoring of Achilles tendinopathy. Collagen hybridization can also be exploited to deliver therapeutics for site-specific and sustained treatment of tendinopathy (*49*). Meanwhile, CHP-based live imaging and histopathology can be used in animal models to validate emerging quantitative MR techniques, such as ultrashort echo time (UTE) MRI [e.g., UTE-T2* mapping and UTE-magnetization transfer (UTE-MT)], which reflects the structural change in tendon through measuring changes in matrix-bound water content (*42*, *64*). In combination, these techniques will establish a correlation between the level of collagen damage in tissue and the MRI signal characteristics, allowing for noninvasive monitoring of tendon overuse and early detection of not only Achilles tendinopathy but also a variety of musculoskeletal diseases and injuries.

## MATERIALS AND METHODS

### Animals

All animal experiments were approved by the animal ethics committee of the Fifth Affiliated Hospital of Sun Yat-sen University (00095) and carried out in accordance with the regulations of the committee. All mice and rats were purchased from specific pathogen–free Biotechnology (Beijing). The animals were kept in a temperature-controlled environment with a 12-hour light/12-hour dark cycle and free access to tap water and normal food pellets. Male SD rats (8 to 10 weeks old, weight 200 to 280 g) were used for the treadmill exercises. All animal studies were summarized in table S1.

#### One-time treadmill running

Thirty SD rats were randomly divided into five groups (*n* = 6): 0 hours p.r., 0 hours (3) p.r., 12 hours p.r., 7 days p.r., and the cage control group. Rats in the running groups ran for 1 hour on a treadmill (KW-PT, Nanjing Calvin Biotechnology) at a 10° incline at a speed of 20 m/min except for the 0-hour (3) p.r. group, in which the rats ran for 3 hours on the treadmill at the same speed. Before running, the rats were trained for 10 min at a progressively increasing speed from 12 to 20 m/min (*37*). The running behavior was prompted by airflow and a mild electric stimulus at the rear of each treadmill chamber (fig. S1B). Airflow was applied to all rats to urge running throughout the tests. To rats that stepped off of the belt, mild electric stimulation was applied at the rear end of the treadmill to make them return to running. At 0 hours (immediately), 12 hours, or 7 days p.r., the rats were euthanized, and their Achilles tendons were harvested (Fig. 1D and tables S1 and S2). Rats in the control group maintained normal activity without running throughout the duration of the study until they were euthanized at the same time as the 12-hour p.r. group. To better observe changes in the Achilles tendon during the rest phase after running, 16 SD rats were divided into four groups (*n* = 4): 12 hours p.r., 2 days p.r., 4 days p.r., and 7 days p.r.

#### Long-term treadmill running

Twenty animals were randomly divided into four groups (*n* = 5): the 4-week running group, 12-week running group, 4-week control group, and 12-week control group. Rats in the running group ran for 1 hour per day, 5 days per week, for 4 or 12 weeks on the treadmill with a 10° incline. During week 1, the duration and pace of running were gradually increased until the rats could run for 1 hour at 17 m/min (table S5). From week 2, the speed was set at 20 m/min (*37*). Rats in the cage control group maintained normal cage activity throughout the duration of the study. The rats of the running and control groups were euthanized after week 4 or 12, and their Achilles tendons were harvested (Fig. 5A). To minimize the immediate effects of running, all rats were euthanized at the same time 24 hours after the running group finished the last 1-hour running session.

#### Collagenase-induced Achilles tendon injury models

C57BL/6 mice (male, 8 to 10 weeks old, approximately 23 g in weight) were used for the collagenase-induced injury model. Type I collagenase (100 mg, 256 U/mg; C8140, Solarbio) was dissolved into 50 ml of TESCA buffer solution (pH = 7.4, G0150, Solarbio). Ten microliters of collagenase (about 5.3 U) was subcutaneously injected into the middle area of the mice’s left Achilles tendons with a microsyringe, and 10 μl of PBS was injected into the right Achilles tendon. The injections were repeated daily for 3 days, and the collagenase-induced injury model was developed 30 days after the last injection (*49*, *50*). The mice were injected with Cy5-CHP and imaged with an IVIS Spectrum imager (PerkinElmer Lumina III) 2-hours after injection on day 33. To exclude the difference among rodent species, six rats (male, 8 to 10 weeks old) with an average weight of 250 g were subcutaneously injected with 60 μl of collagenase I solution (about 30 U) in the left Achilles tendon every 2 days for a total of seven injections, and the collagenase-induced injury model was established at day 14 since the first injection. Meanwhile, 60 μl of PBS was injected into the right Achilles tendon of the same rats (*51*).

### In vitro tensile test

Twelve Achilles tendons were harvested from male SD rats (8 to 10 weeks old) and randomly divided into three groups. The tensile test was carried out using the BoseAT-3220 system (Bose, Electro Force Systems Group); each specimen was fixed to the clamps to ensure that the longitudinal axis of the specimens was in line with the axis of the pull-force arm. Before the test, a 0.03-N preload was applied to remove slack. Four samples were loaded to failure to determine the failure strain of the tendons at a rate of 0.05% of strain. One sample of Achilles tendon was excluded from the study because it ruptured where the claw grasped the muscle. Subsequently, eight Achilles tendon samples were divided into two groups to perform the tensile experiment at a strain rate of 0.05%/s to 20.4 and 27.3% strain (approximately 60 and 80% of the estimated failure strain). The force-displacement data were recorded.

### Peptide and probe synthesis and labeling

The CHP probes were synthesized, fluorescently labeled with sulfo-cyanine 5 (Cy5) and purified by high-performance liquid chromatography (HPLC) according to the previously reported methods (*65*, *66*). The purified peptide products were lyophilized and verified by a Shimadzu 8020 matrix-assisted laser desorption/ionization–time-off-light mass spectrometer. In this study, the Cy5-CHP used in all in vitro tissue staining experiments had a sequence of Cy5-Ahx-(GPO)_9_ (O: (2S,4R)-4-hydroxyproline, Ahx: aminohexanoic acid, MALDI-MS, calcd 3160.4 [M + Na]^+^, observed 3160.4 [M + Na]^+^); the Cy5-CHP used in all in vivo targeting experiments had a sequence of Cy5-Ahx-(GfO)_9_ (f: (2S,4S)-4-fluoroproline, MADLI-MS, calcd 3343.3 [M + Na]^+^, observed 3343.8 [M + Na]^+^). The (GfO)_9_ sequence was used due to its ability to stay as single strands at body temperature (*67*). The sequence of the noncollagen-hybridizing control peptide was Cy5-Ahx-OfGGOfGfGfOfOGOfGOOfGGOOffG (Cy5-Ahx-^S^CHP with a scrambled CHP sequence, MADLI-MS, calcd 3343.3 [M + Na]^+^, observed 3343.6 [M + Na]^+^).

The MR probe Gd_*n*_-Cy5-CHP (or Gd_*n*_-Cy5-^S^CHP) was prepared by synthesizing the Cy5-labeled sequence [NH_2_-(AhxK)_2_K]_2_KK(Cy5)-Ahx-(GfO)_9_ on resin before trifluoroacetic acid cleavage and HPLC purification (linear gradient: 5 to 35% acetonitrile in 30 min). The purified peptide precursor was conjugated to 40 equivalent p-SCN-Bn-DOTA (Macrocyclics, B-205) in dimethyl sulfoxide and purified by HPLC. The DOTA-conjugated peptide was mixed with excess GdCl_3_ (Aladdin) in a sodium acetate solution and purified by ultrafiltration [Vivaspin turbo 15 PES, molecular weight cutoff (MWCO): 3000, Sartorius] to obtain the final probe product Gd_*n*_-Cy5-CHP or the sequence-scrambled control probe Gd_*n*_-Cy5-^S^CHP.

### Histology and microscopy

All staining was performed on cryosections with a thickness of 8 μm. The sections were washed with PBS to remove the optimal cutting temperature (OCT) compound before staining. H&E staining was performed according to the manufacturer’s instructions (Phygene, PH0516).

#### Immunohistochemistry

Following blocking against endogenous peroxidases and nonspecific stains (MXB, KIT-9707, 10 min for each step) and washing, tendon sections were stained with an anti–collagen I (2 μg/ml; Abcam, ab34710) or anti–collagen III antibody (1 μg/ml; Boster, A00788-3) overnight at 4°C. After a thorough wash with PBS, each section was treated with a biotinylated goat anti–rabbit–immunoglobulin G (IgG) (10 min; MXB, KIT-9707) and a streptavidin–horseradish peroxidase conjugate (10 min; MXB, KIT-9707) before color development with 3′3-diaminobenzidine (MXB, DAB-0031). The H&E and immunohistochemistry (IHC) stains were imaged on an EVOS M7000 (Thermo Fisher Scientific) microscope using a 20× or 40× objective lens.

#### Immunofluorescence

Following blocking with 10% goat serum for 1 hour at room temperature, the tendon sections were stained with an anti-MMP1 (2 μg/ml; GeneTex, GTX100534), anti-MMP9 (2 μg/ml; Abcam, ab38898), anti-MMP13 (3 μg/ml; Proteintech, 18165-1-AP), anti–α-SMA antibody (1 μg/ml; Abcam, ab124964), anti-IL-6 antibody (2 μg/ml; Affinity, DF6087), or anti-TNMD antibody (2 μg/ml; Bioss, bs-7525R) overnight at 4°C. After a thorough wash with PBS, each section was treated with an Alexa Fluor 488–labeled goat anti–rabbit-IgG (4 μg/ml; GST, 4412S) for 1 hour at room temperature, followed by washing in PBS and DAPI staining (1:1000 dilution in PBS; Beyotime, C1002) for 20 min at room temperature. The images were scanned on the EVOS M7000 (light cubes: GFP and DAPI, 10× objective). The DAPI-stained cell nuclei were imaged by a confocal laser scanning microscopy (Zeiss LSM880) with a 60× objective.

#### CHP staining

Before staining, Cy5-CHP (5 μM in 2 ml of PBS) was heated to 80°C to dissociate the peptide into unfolded single strands and rapidly cooled down to room temperature on ice (1 min) (*5*) before being used to stain the sections overnight at 4°C. After PBS wash and DAPI staining, we performed the immunofluorescence staining after the CHP stain was complete. The slides were imaged with the EVOS M7000 (light cubes: Cy5 and DAPI, 10× objective).

The fluorescence and IHC images were analyzed using the ImageJ software (Fiji 2.14.0) by manually thresholding the images (detailed in the Supplementary Materials). The numbers of cell nuclei with different levels of circularity were quantitatively analyzed using the “Analyze Particle” function of ImageJ and counted from 12 fields of view for each group (Fig. 3B and fig. S12B) [Circularity: 4 × pi × (area/perimeter^2^), defined as the normalized ratio of the area to the perimeter with a circle having a value of 1 and a line having a value of 0].

#### Scanning electron microscope

The obtained Achilles tendon tissues were fixed overnight in a 4% (v/v) paraformaldehyde (PFA) solution. After washing with water for 1 hour, the samples were dehydrated using solutions with increasing concentrations of *t*-butanol (TB) with 15 min in each solution. The tendon tissues were fixed with a conductive adhesive (NEM-005, Nisshin EM) and sputter-coated with gold for imaging with SEM (high voltage: 10 kV, magnification: ×17,500; phenom pure, Thermo Fisher Scientific).

#### SHG microscopy

An upright laser-scanning multiphoton microscope (FVMPE-RS, Olympus) was used to perform SHG microscopy on the cryosections (8 μm thick) of Achilles tendons. To acquire the SHG signals from collagen, the slides were imaged with 860-nm excitation and detection in 420 to 441 nm. The disrupted fiber orientation in SHG images was quantitatively analyzed using the “OrientationJ” plug-in of the ImageJ software (Supplementary Materials).

### Trypsin-hydroxyproline testing

The trypsin-hydroxyproline test was performed according to a previously reported protocol (*5*). We extended the trypsinization to 2 days and added preservatives [1 mM iodoacetamide (Solarbio) and pepstatin A (10 μg/ml; Glpbio)] to the digestion buffer to ensure that the Achilles tendons were sufficiently digested. The trypsin-solubilized collagen (supernatant) and the remaining undigested tissues were separately hydrolyzed in hydrochloric acid at 110°C. The hydroxyproline content of each solution was measured by a hydroxyproline assay kit (Acmec, BC0250) according to the manufacturer’s instructions. The absorbance of the final solution was read in a 96-well plate at 560 nm using a microplate reader (BioTek, Synergy HTX). The percentage of denatured collagen solubilized from each sample was calculated by dividing the amount of hydroxyproline in the sample’s digestion solution by the total amount of hydroxyproline in both the undigested remaining and its digestion solution.

### RNA-seq and bioinformatic analysis

Rats in the running group were euthanized immediately (0 hours) or 12 hours after 1 hour of treadmill running. Fresh Achilles tendon tissues from the right legs of the running and control rats were immediately dissected and placed in RNALater (Beyotime) to prevent RNA degradation. The total RNA was extracted after the tissues were mechanically pulverized in TRIzol (Thermo Fisher Scientific). After verification of the RNA integrity by Qsep100 (BIOptic; RNA integrity number > 7.0), polyadenylate mRNAs were purified from total RNA using mRNA capture beads (Vazyme, N401). Then, the mRNA was reverse-transcribed to create a cDNA library. VAHTS Stranded mRNA-seq Library Prep Kit for Illumina V2 (Vazyme Biotech, NR612-02) was used for library preparation. RNA-seq was performed using the Illumina NovaSeq 6000 sequencer at Guangzhou Epibiotek. Reads were aligned to the rat Ensemble genome Rnor_6.0 using Hisat2 aligner (v2.1.0) under parameters: “--rna-strandness RF”. Read counts and gene length were calculated using featureCounts (v1.6.3). Normalization and differentially expressed genes (DEGs) analysis was conducted using DESeq2 v1.30.1. Genes were identified as differentially expressed with a fold change ≥ 2 and false discovery rate adjusted *P* value < 0.05. Gene expression heatmaps were generated using the ComplexHeatmap R package (*68*). We performed GO enrichment analysis for all differentially expressed genes, applying the R package clusterProfiler (v3.6.0) (*69*). To find enriched GO terms, an over-representation test based on the hypergeometric distribution of the GO terms was performed. The biological processes ontology term option was selected, and the *P* value of each pathway was calculated and adjusted using the Benjamin-Hochberg method.

### In vivo fluorescence imaging

After being anesthetized by isoflurane (2 to 3% mixed with air for mice, 3 to 5% for rats; R510-22, RWD Life Science), the animals were prepared for probe injection and imaging starting from skin depilation on both legs. Cy5-CHP (0.02 nmol/μl in PBS) was subcutaneously injected into the peritendinous tissue of the two Achilles tendons (5 μl per leg for rats and 2 μl per leg for mice). We performed fluorescence imaging on the mice 1 month after the peritendinous collagenase injections when a disease phenotype and histopathological feature mimicking human Achilles tendinopathy were developed (Fig. 6A) (*49*). The animals were imaged with an IVIS Spectrum imager (PerkinElmer Lumina III) 2 hours after injection under the following settings: excitation/emission wavelengths (Ex/Em): 620/670 nm, pixel: 4, exposure time: 1 s, visual field: D (for rats) and B (for mice). The total radiation efficiency of each Achilles tendon was quantitatively measured using the Living Image software (PerkinElmer) after circling the area of interest with the same size on each ankle.

### Light-sheet fluorescence microscopy

After subcutaneous injection of Gd_*n*_-Cy5-CHP and in vivo imaging, the Achilles tendons of the rats or mice were collected and cleared in the dark (*66*, *70*, *71*). After being fixed in 4% PFA at room temperature for 24 hours, the samples were washed with PBS for 30 min before decolorizing with Quadrol (25% v/v in water; Sigma-Aldrich, 122262) for 1 day. After being washed three times with PBS (1 hour each round), the samples were delipidated with TB (Sigma-Aldrich, 360538) [30, 50, and 70% (v/v) TB in water] and dehydrated with a TB–polyethylene glycol (PEG) solution made by 70% (v/v) TB, 27% (v/v) PEGMEMA500 (Sigma-Aldrich, 447943), and 3% (w/v) Quadrol for 2 days. Last, the samples were immersed in a clearing medium benzyl benzoate-PEG (BB-PEG) made by 75% (v/v) benzyl benzoate (Sigma-Aldrich, W213802), 22% (v/v) PEGMEMA500, and 3% (w/v) Quadrol for at least 1 day. The cleared Achilles tendon specimens were scanned unilaterally using a LaVision Biotec Ultramicroscope II equipped with an scientific complementary metal-oxide semiconductor camera and a 4× objective lens that was immersed in BB-PEG medium in the imaging chamber (Ex/Em: 630/680, attenuator: 15%, scanning layer thickness: 4 μm, optical film width of 70%). The 3D images acquired by ImSpector (LaVision BioTec) were reconstructed with the Imaris software using the blend algorithm to produce movies at a frame rate of 25 fps.

### In vivo MR imaging of collagenase-injured Achilles tendon

One month after the collagenase injections, when an Achilles tendinopathy phenotype mimicking human disease was developed histologically (Fig. 7A) (*49*), MR imaging was carried out on five mice in a 9.4-T small animal MRI scanner (Bruker BioSpec94/30 USR) with a ^1^H planar receive-only surface coils (inner diameters, 20 mm; Bruker) using a T1-weighted rapid acquisition with relaxation enhancement (T1-RARE) sequence. Mice were anesthetized under isoflurane (2 to 3% mixed with air) only during agent injections and MR scans. Both Achilles tendons of each mouse were prescanned before probe injection. Then, Gd_*n*_-Cy5-^S^CHP was subcutaneously injected into the diseased Achilles tendon area (50 nmol in 10 μl of PBS), and the T1-weighted scan was performed 6 hours after injection. After another week, the diseased and normal legs of the same mouse both received subcutaneous injection of Gd_*n*_-Cy5-CHP (50 nmol per leg in 10 μl of PBS) in the Achilles tendon areas and were scanned before and 6 hours after injection. Main imaging parameters: T1-FS: echo time (TE) = 6.1 ms; repetition time (TR) = 400 ms; slice thickness: 0.3 mm; field of view (FOV) = 20 × 20 mm; matrix dimensions = 256 × 256; bandwidth = 434.0 kHz; and echo train length (ETL) = 2; scanning time was 10 min 14 s. T2-FSE: TE = 30.0 ms; TR = 1500 ms; slice thickness: 0.3 mm; FOV = 20 × 20 mm; matrix dimensions = 228 × 228; bandwidth = 219.3 kHz; ETL = 8. The MR images were processed and analyzed by the RadiAnt DICOM Viewer software.

### Data and statistical analysis

The experimental data were statistically analyzed by GraphPad software (version 8.0.0, Dotmatics), and the quantitative data were expressed by means ± standard error of the mean. Unless otherwise noted, one-way analysis of variance (ANOVA) and Tukey’s multiple comparisons test were used. *P* values below 0.05 were regarded as statistically significant, and those above 0.05 were not shown in any figure. The correlation of MMP and CHP fluorescence changes was interpreted by the Pearson correlation coefficient (*r*) and tested with the null hypothesis *H*_0_ = true correlation between the groups is 0. An *r* value closer to 1 and a smaller *P* value suggest a strong correlation between the datasets. Chi-square analysis was used to test whether the distribution differs between the circularity of nuclei in each group in the one-time treadmill running tests. The T1 signal differences in MR detection were analyzed using a paired *t* test.

## Supplementary Materials

### The PDF file includes:

Supplementary Text Figs. S1 to S21 Tables S1 to S6 Legends for movies S1 to S3 Legend for data s1
