# Supplementary material for: Collagen denaturation in post-run Achilles tendons and Achilles tendinopathy: In vivo mechanophysiology and magnetic resonance imaging
Source: Sci Adv. 2024 Oct 2;10(40):eado2015. doi: 10.1126/sciadv.ado2015 (PMC11446262; doi:10.1126/sciadv.ado2015)
Supplement: Supplementary file 1 — Supplementary Text Figs. S1 to S21 Tables S1 to S6 Legends for movies S1 to S3 Legend for data s1 [file sciadv.ado2015_sm.pdf]

Science Advances
AAAS

Supplementary Materials for

Collagen denaturation in post-run Achilles tendons and Achilles tendinopathy: In vivo mechanophysiology and magnetic resonance imaging

Yijie Fang *et al.*

Corresponding author: Yang Li, liyang266@mail.sysu.edu.cn; Shaolin Li, lishlin5@mail.sysu.edu.cn; Man Li, liman26@mail.sysu.edu.cn; Yijie Fang, fangyj5@mail.sysu.edu.cn

Sci. Adv. **10**, eado2015 (2024)
DOI: 10.1126/sciadv.ado2015

The PDF file includes:

Supplementary Text
Figs. S1 to S21
Tables S1 to S6
Legends for movies S1 to S3
Legend for data S1

Other Supplementary Material for this manuscript includes the following:

Movies S1 to S3
Data S1

Supplementary Text

SUPPORTING METHODS

Gene expression verification with qRT-PCR

We verified the transcription of four genes in the rat Achilles tendons following 1-hour treadmill running: *Mmp1* (tnt5741b, BioTNT), *Mmp9* (tnt5396a, BioTNT), and *Mmp13* (tnt5742b, BioTNT) using qRT-PCR. Fresh Achilles tendon tissues from the right legs of the running and control rats were immediately dissected and placed in RNALater™ (Beyotime) to prevent RNA degradation. The total RNA was extracted after the tissues were mechanically pulverized in TRIzol (Thermo Scientific). The total RNA was reverse-transcribed to cDNA (cDNA first strand synthesis RNA reverse transcription reagent, A2010A0601, BioTNT). The quality and quantity of RNA were checked with a NanoDrop spectrophotometer (Thermo Fisher) before conversion. qRT-PCR was performed in an Applied Biosystems 7500 Fast Real-Time PCR System (Thermo Fisher) with the SYBR-Green I quantitative polymerase chain reaction kit (A2010A012, BioTNT). *Gapdh* (tnt0274e, BioTNT) was used as the reference gene. Primer information is provided in Table S6.

Image analysis using ImageJ

Fluorescence quantification. Fluorescence quantification was performed from grayscale images (16-bit) of fluorescence images, typically scans of full-size Achilles tendon sections from an EVOS microscopy. Images of the tendon tissues were manually thresholded for fluorescence signal. The same thresholding parameters were used for each fluorescence marker throughout the entire study for consistent quantification (see below). Integrated density was used as the output value for quantitative and statistical analyses.

	**CHP**	**CHP**	**CHP**	**MMP1**	**MMP9**	**MMP13**	**α-SMA**
**Experiment**	Fig. 1C	fig. S3 only	Throughout the study except Fig. 1C and fig. S3	Fig.1 and Fig. 5			Fig. 3
**Imaging parameters**	Cy5 channel: Light, 25%; Exposure, 0.2 s; Gain, 1	Cy5 channel: Light, 25%; Exposure, 0.2 s; Gain, 1	Cy5 channel: Light, 50%; Exposure, 0.5 s; Gain, 1	GFP channel: Light, 50%; Exposure, 1 s; Gain, 3	GFP channel: Light, 50%; Exposure, 1 s; Gain, 3	GFP channel: Light, 50%; Exposure, 1 s; Gain, 6	GFP channel: Light, 50%; Exposure, 1 s; Gain, 1
**Thresholding parameters in ImageJ**	Min: 1012; Max: 4095	Min: 1000; Max: 4095	Min: 1413; Max: 4079	Min: 514; Max: 4079	Min: 1220; Max: 4079	Min: 626; Max: 4079	Min: 710; Max: 4079
**Note**	Mechanical damage	Mechanical versus *in vivo* damage	*In vivo* damage				

Quantification of damaged fiber orientation. The ImageJ plug-in "OrientationJ" was used to quantify the orientation of the disorganized collagen fibers. This image analysis program analyzes pixel gradients in both the vertical and horizontal dimensions to determine each pixel's local orientation and isotropic features. The dominant orientation of the injured fibers was measured using the built-in "Dominant Direction" tool. For every

image, a hue-saturation-brightness (HSB) color-coded map and distribution plot of fiber orientations were produced using the "Distribution" function. The distribution plots count the total number of pixels with a defined orientation (one-degree resolution). To provide a good comparison of the alterations in collagen fiber distribution between the groups, the dominant direction of collagen fiber in the Achilles tendon was adjusted to 0° on the distribution plots of fiber orientations.

IHC image quantification. Quantitation of type I and III collagen IHC was conducted in a blinded fashion with the ImageJ Software using the "IHC Profiler" plug-in. An area with a certain level of staining coloration was graded as High positive (3), Positive (2), Low Positive (1), and Negative (0) based on its gray value: High Positive (gray value 0-60), Positive (gray value 61-120), Low Positive (gray value 121-180), and Negative (gray value 181-236). The percentage area size under each coloration grade was multiplied by its grade number to calculate the total "H-score" (max: 300, min: 0) of the whole image.

Measurements of the T1 relaxivity of the MR probes.

The number of Gd^{3+} per CHP probe molecule was estimated to be 6.3 using inductively coupled plasma-optical emission spectrometers (iCAP™ Q, Thermo Fisher). T1 relaxivity of our MR probes was measured by a 9.4 T small animal MRI scanner (Bruker BioSpec94/30 USR) with ^{1}H planar receive-only surface coils (inner diameters 20 mm, Bruker) at room temperature. Probes Gd_n-Cy5-CHP and Gd_n-Cy5-^{S}CHP were prepared into 200 µL solutions with Gd^{3+} concentrations of 14, 28, 56, and 112 µM in water and scanned along with a serial dilution of Magnevist (Gadopentetate Dimeglumine). To obtain T1-weighted images, T1 mapping sequences with the following parameters were employed: echo time (TE) = 7.0 ms; repetition time (TR) = 5500, 3000, 1500, 800, 400, and 200 ms; slice thickness: 0.5 mm; Field of View (FOV) = 30 × 25 mm; matrix dimensions = 128 × 128; bandwidth = ± 610.4 kHz; and echo train length (ETL) = 2; scanning time was 12 min 9 s. Plots of 1/relaxation time (1/T1, s^{-1}) versus Gd^{3+} and CHP concentrations were constructed to obtain relaxivity values (r_1) as their slopes.

***In vitro* MR and fluorescence imaging of the rat Achilles tendons.**

Nine rat Achilles tendon samples were heat denatured in boiling water for 1 min (to fully denature the collagen as positive controls) and divided into 3 groups to be incubated in 5 mL of PBS, Gd_n-Cy5-CHP, and Gd_n-Cy5-^{S}CHP, respectively (n = 3, probe concentration: 5 µM) at 4 °C for at least overnight. Following extensive wash with PBS, the samples were fluorescently imaged with an IVIS Spectrum imager (PerkinElmer Lumina III, Ex/Em wavelength: 620/670 nm, pixel: 4, exposure time: 1 s, field of view: A) and a 9.4 T MRI scanner (Bruker BioSpec94/30 USR) with a ^{1}H planar receive-only surface coils (inner diameters 20 mm, Bruker) using a T1-weighted rapid acquisition with relaxation enhancement (T1-RARE) sequence (TE = 6.0 ms; TR = 200 ms; slice thickness: 0.5 mm; ETL = 2; FOV: 20 × 20 mm; matrix dimensions: 136 × 136; scanning time was 2 min 16 s).

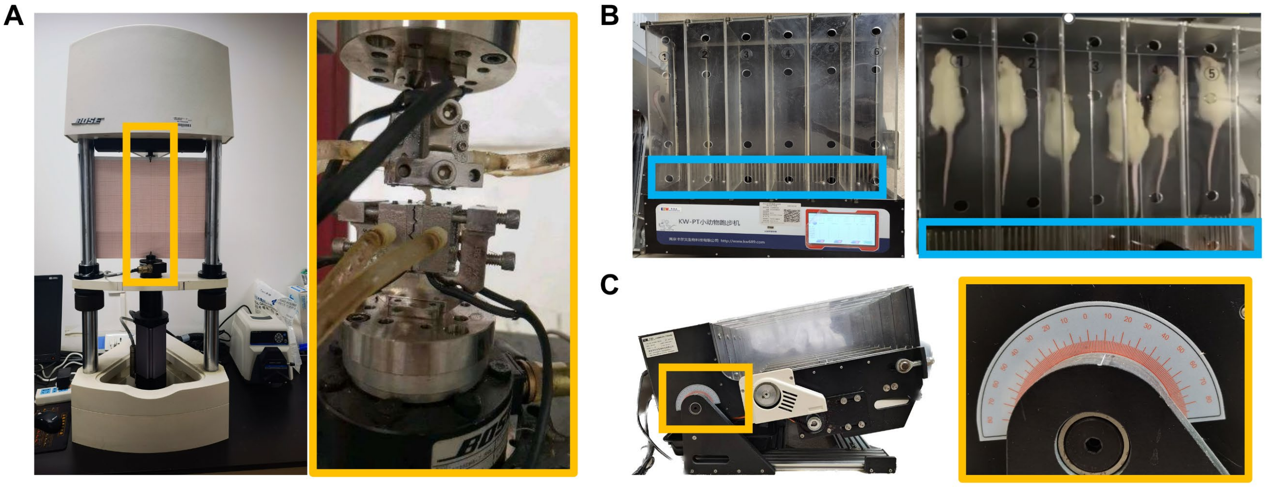

Fig. S1. Instruments for the *in vitro* mechanical tensile testing and rat treadmill running. (A) Using a BoseAT-3220 system (Bose, Electro Force Systems Group), rat Achilles tendons were mounted with clamps and loaded with an increase of 0.05% strain per sec. **(B)** In our study, the rats ran on a treadmill (KW-PT, Nanjing Calvin Biotechnology) set at a 10° incline **(C)** in six runways (see Movie S1). The treadmill was equipped with an electric grid (0-0.5 mA, blue box) and airflow for running stimulation.

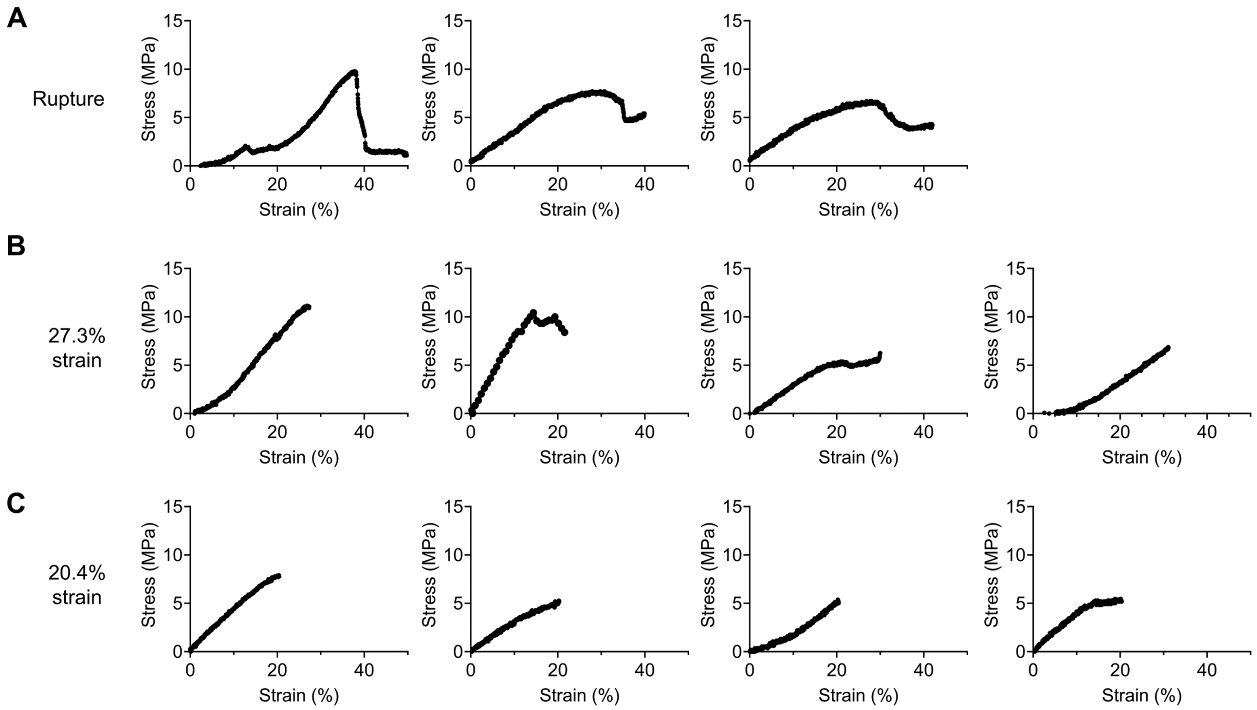

Fig. S2. The *in vitro* mechanical tensile testing of rat Achilles tendons. Stress-strain curves for the SD rats' Achilles tendons loaded *in vitro* with an increase of 0.05% strain per second until rupture **(A)**, or up to 27.3% **(B)** and 20.4% strain **(C)**, which were approximately 80% and 60% of the estimated failure strain, respectively.

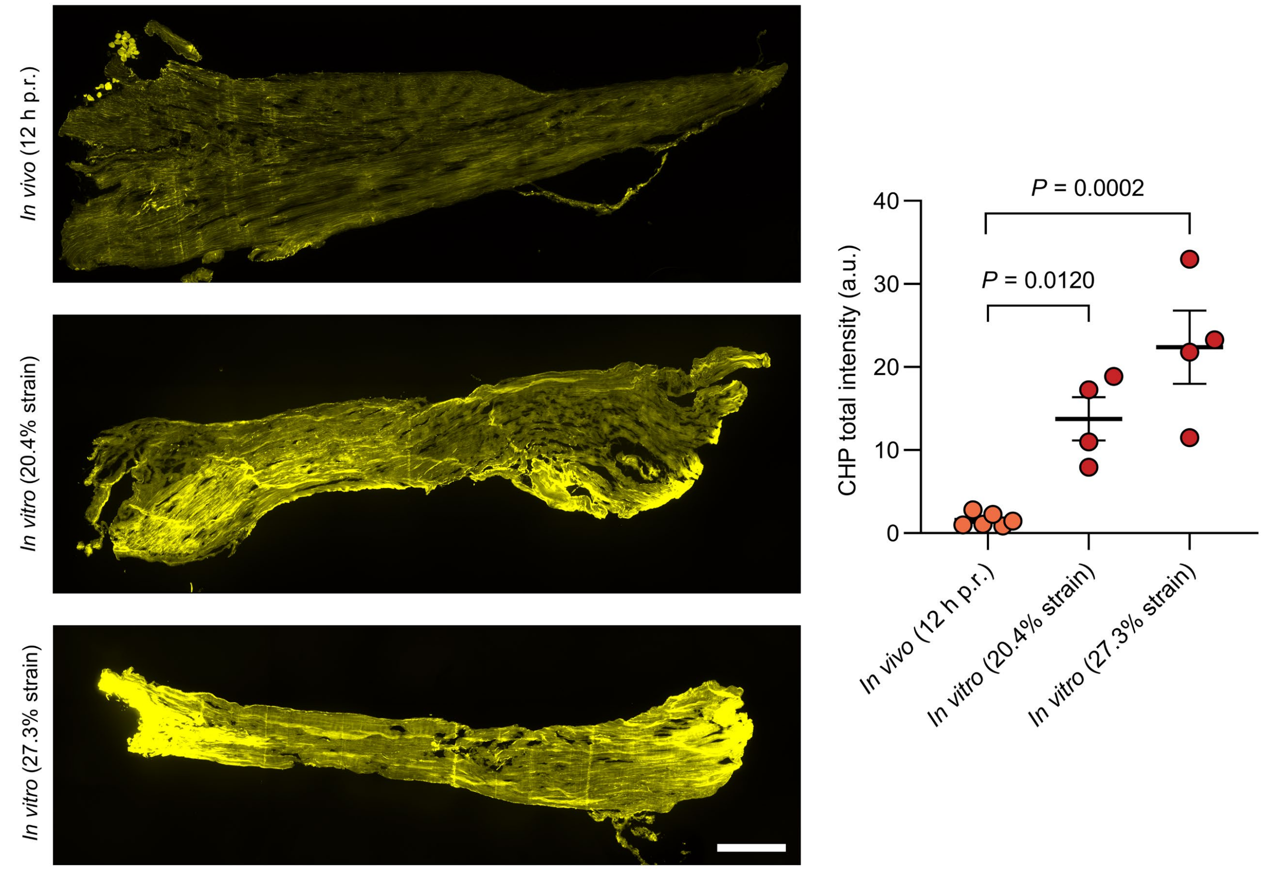

Fig. S3. Comparison between the level of collagen denaturation caused by running (*in vivo*) and tensile loading (*in vitro*). Under identical fluorescence imaging conditions, the Cy5-CHP-stained cryosections of the Achilles tendons showed that the levels of collagen denaturation in the mechanically loaded groups were greater than the 12 h p.r. group over an order of magnitude. Each image was representative of similar results from all the rats within each group. The CHP intensity quantifies the total fluorescence signals from each scan of the whole tissue section. Scale bar: 1 mm. Data were expressed as mean ± s.e.m.; statistical analysis: one-way ANOVA and Tukey.

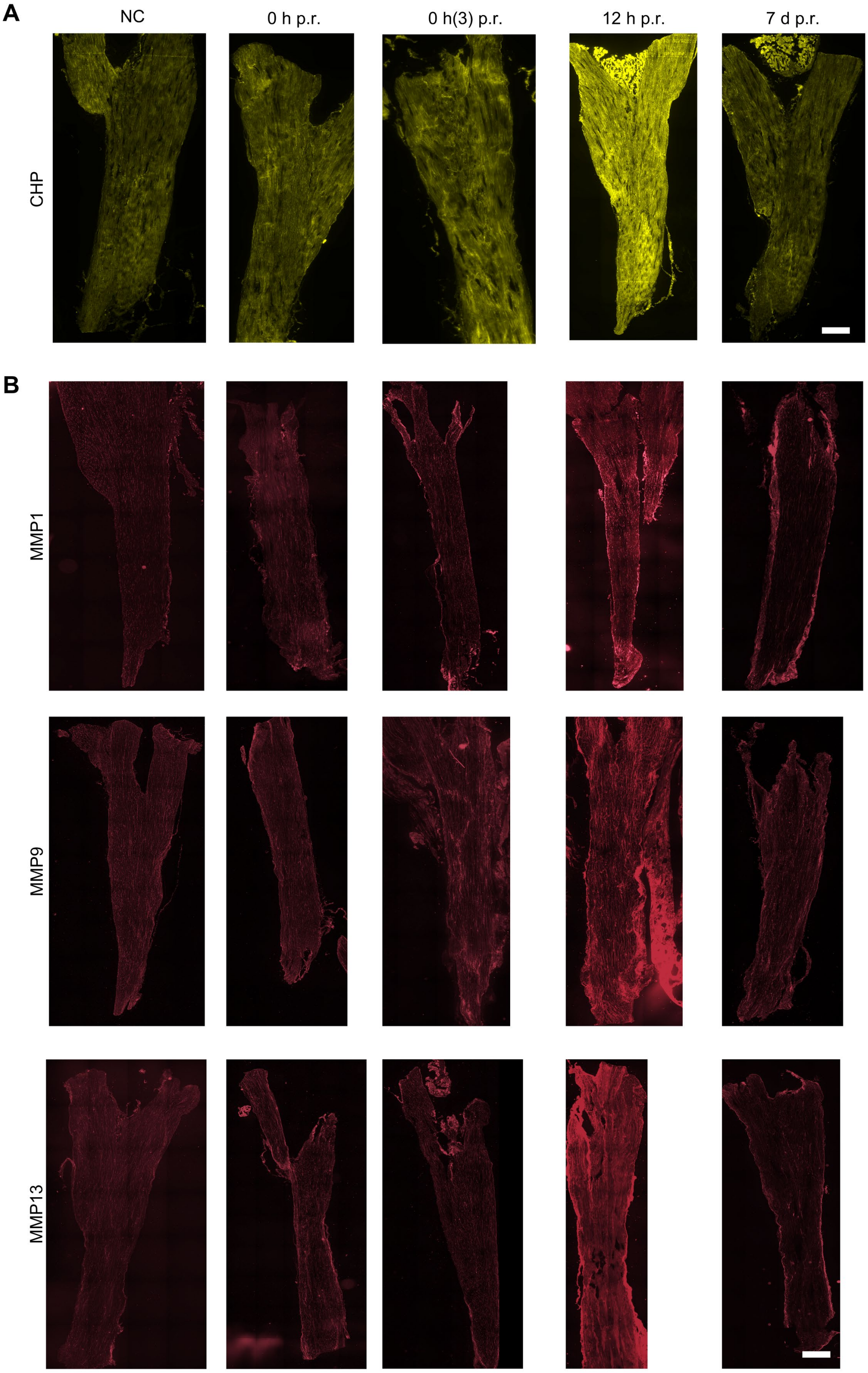

Fig. S4. The CHP and MMP immunostaining of the rat Achilles tendons from the one-time treadmill running experiment. Fluorescence scans of Cy5-CHP staining **(A)** and MMP1, 9, 13 immunofluorescence staining **(B)** of cryosections of the Achilles tendons of the SD rats from the one-time treadmill running test. Each scan was representative of similar results from six rats within each group. Scale bars: 1 mm.

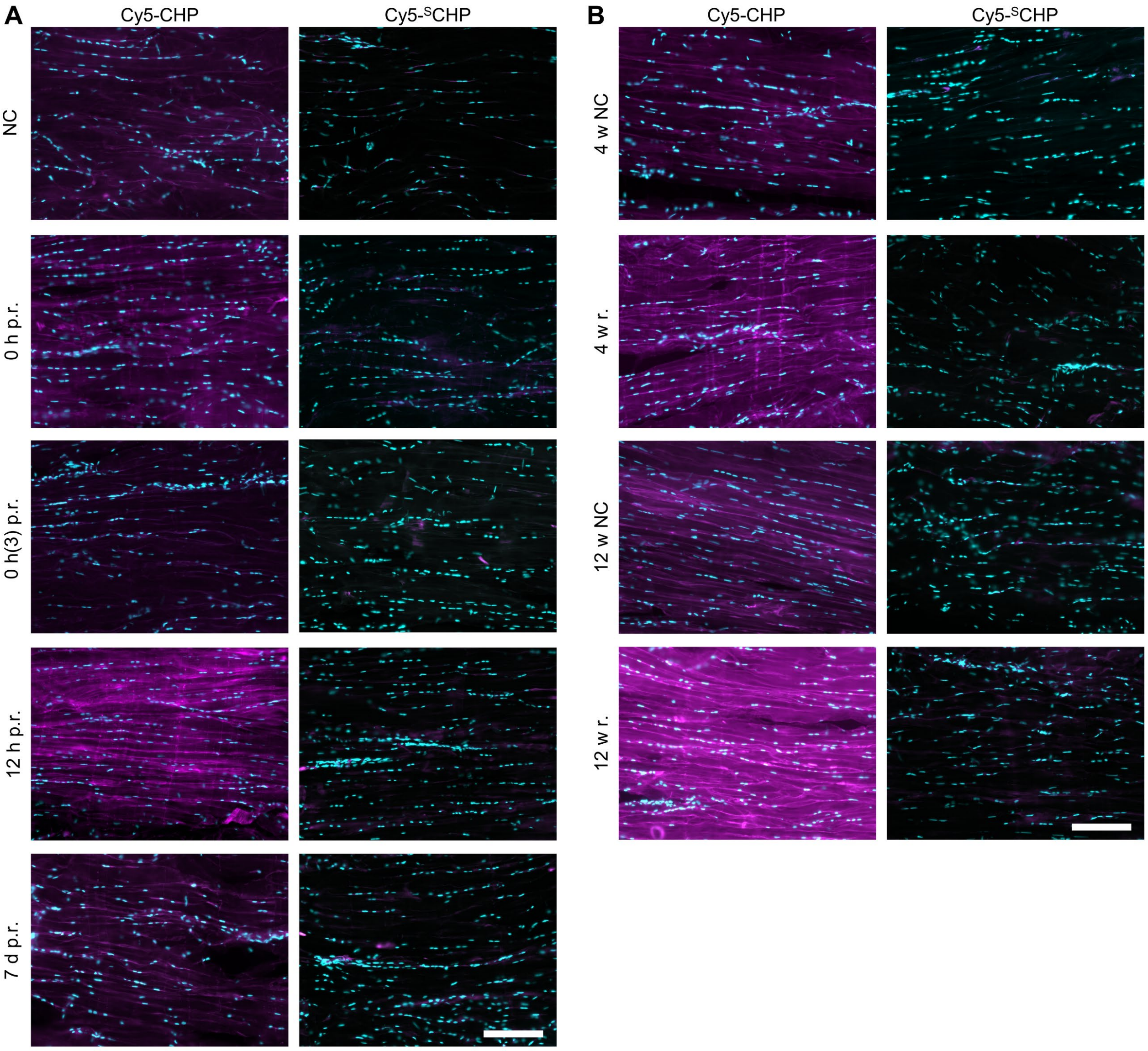

Fig. S5. Validation of the triple-helix hybridization of CHP staining. Fluorescence images of the cryosections of the Achilles tendons from the one-time **(A)** and long-term treadmill running groups **(B)** stained by Cy5-CHP / Cy5-^{S}CHP (magenta) and DAPI (cyan). Denatured collagen was undetectable by our control probe Cy5-^{S}CHP with a scrambled CHP sequence (Cy5-Ahx-PGOGPGPOPOGOGOPPGOOGPGOOPPG, MALDI-MS, calcd 3160.37 $[M+Na]^{+}$, observed 3160.42 $[M+Na]^{+}$). Each image was representative of similar results from six rats within each group. Panels A and B were imaged with different parameters. Scale bars: 75 μm.

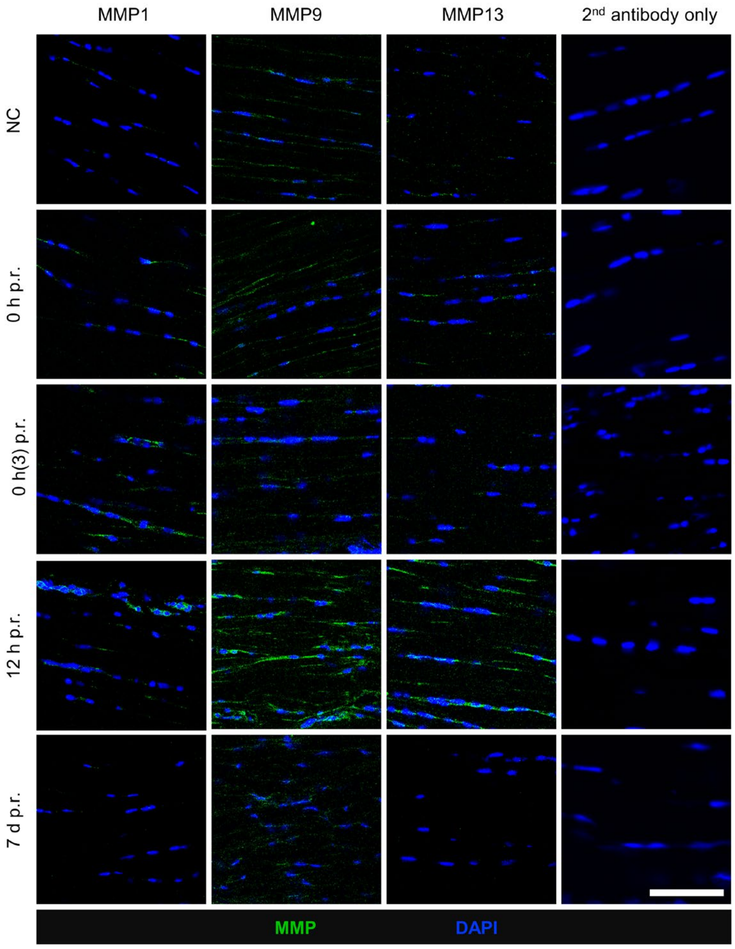

Fig. S6. Validation of the specificity of immunofluorescence against MMP1, 9, 13. Confocal fluorescence microscopy images of the rat Achilles tendon cryosections from the 12 h p.r. group stained with DAPI and anti-MMP antibodies. For the 2nd-antibody-only group, the tissue sections were stained as usual without using the primary MMP antibodies (same as in Fig. 1H); the lack of fluorescence signals in this group demonstrated that the detected fluorescence from the immunostaining of the MMP1, 9, 13 groups is not due to tissue autofluorescence, background fluorescence, or non-specific binding of the 2nd antibody. Scale bar: 75 μm.

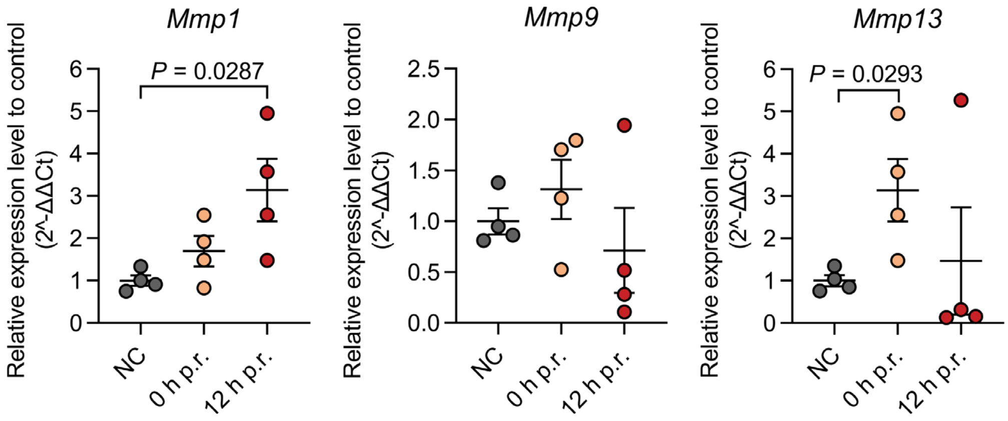

Fig. S7. Quantitative real-time reverse transcription polymerase chain reaction (qRT-PCR) measurement of the *Mmp* gene transcription in the rat Achilles tendons from the one-time treadmill running study. Gene expression for qRT-PCR was normalized to *Gapdh*. Data were presented as mean ± s.e.m. after being normalized to the mean value of the NC group; statistical analysis: one-way ANOVA and Tukey.

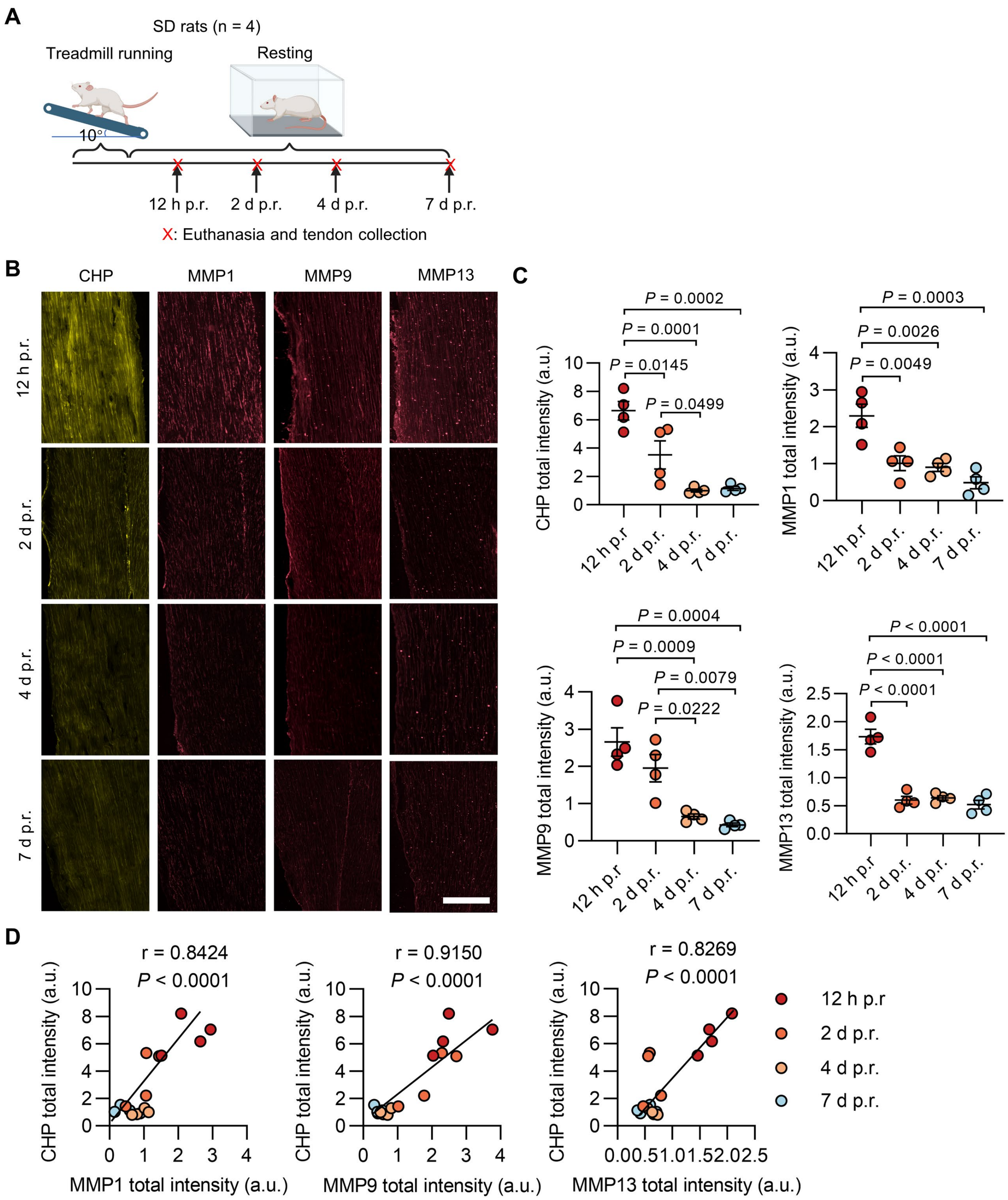

Fig. S8. ***In vivo*** **collagen denaturation in the recovery stage of the Achilles tendons after 1-hour treadmill running. (A)** Study design. Rats were euthanized to collect their Achilles tendons at 12 h, 2 d, 4 d, and 7 d p.r. **(B)** Representative images of the cryosections of the Achilles tendon with Cy5-CHP staining and immunostaining against MMP1, 9, 13 at each time point. **(C)** Quantified total Cy5-CHP, MMP1, 9, and 13 fluorescence intensities of the whole-section scans of the Achilles tendons from each group. **(D)** Correlation analysis between the signals of MMP1, 9, 13 and the CHP signals on cryosections of Achilles tendons, respectively, interpreted by the Pearson correlation coefficient (r). The fluorescence images are representative of similar results from four rats within each group **(B)**. Scale bar: 150 µm **(B)**. Data were expressed as mean ± s.e.m.; statistical analysis: one-way ANOVA and Tukey **(C)**. The fig. S8A was created with BioRender.com.

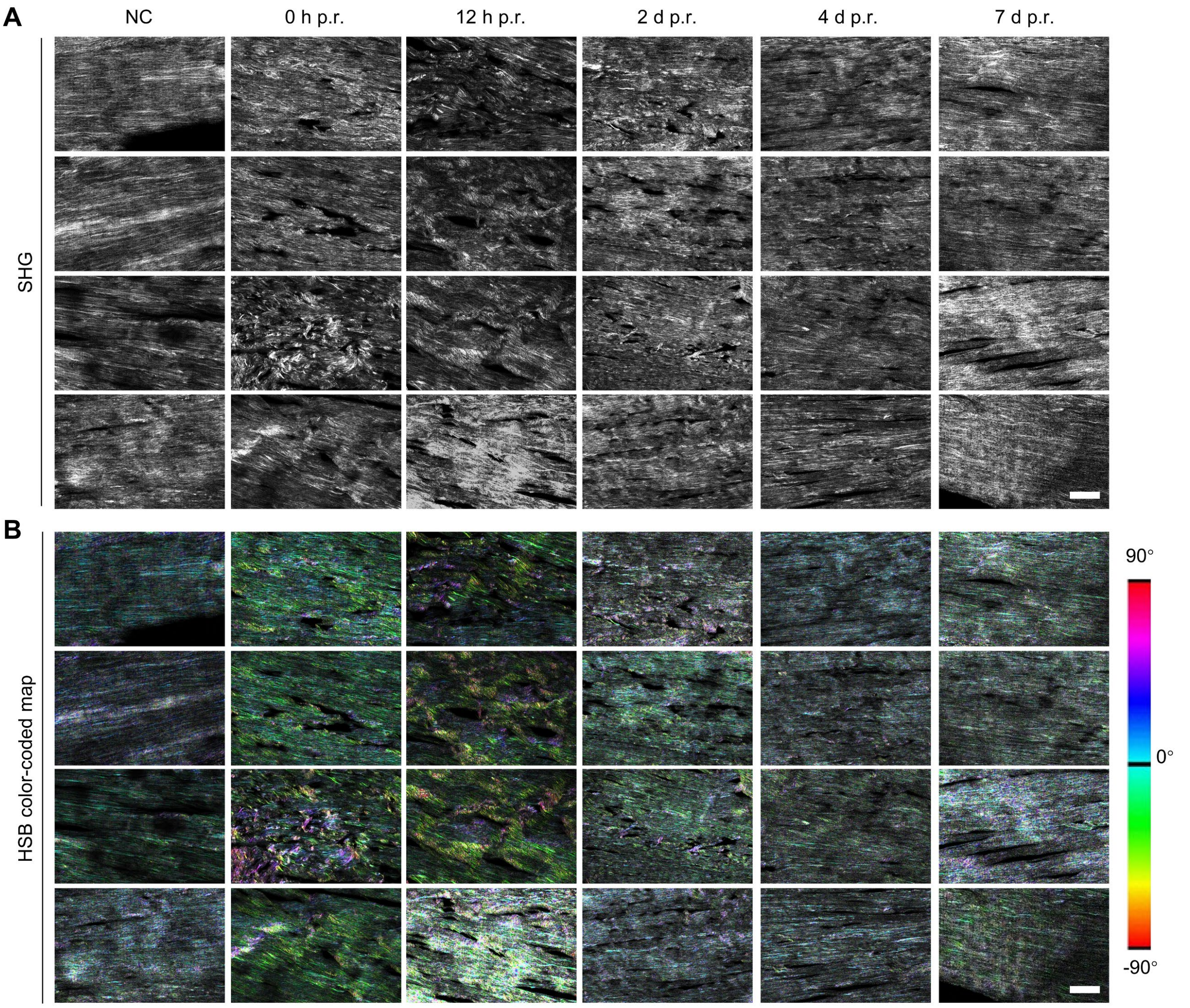

Fig. S9. Additional SHG microscopy images of the organization of the collagen fibers in the post-run rat Achilles tendons. For each group, four SHG images **(A)** were randomly acquired from two tissue cryosections. For pixel orientation visualization, a corresponding HSB color-coded map was generated for each image **(B)**. Scale bars: 100 μm.

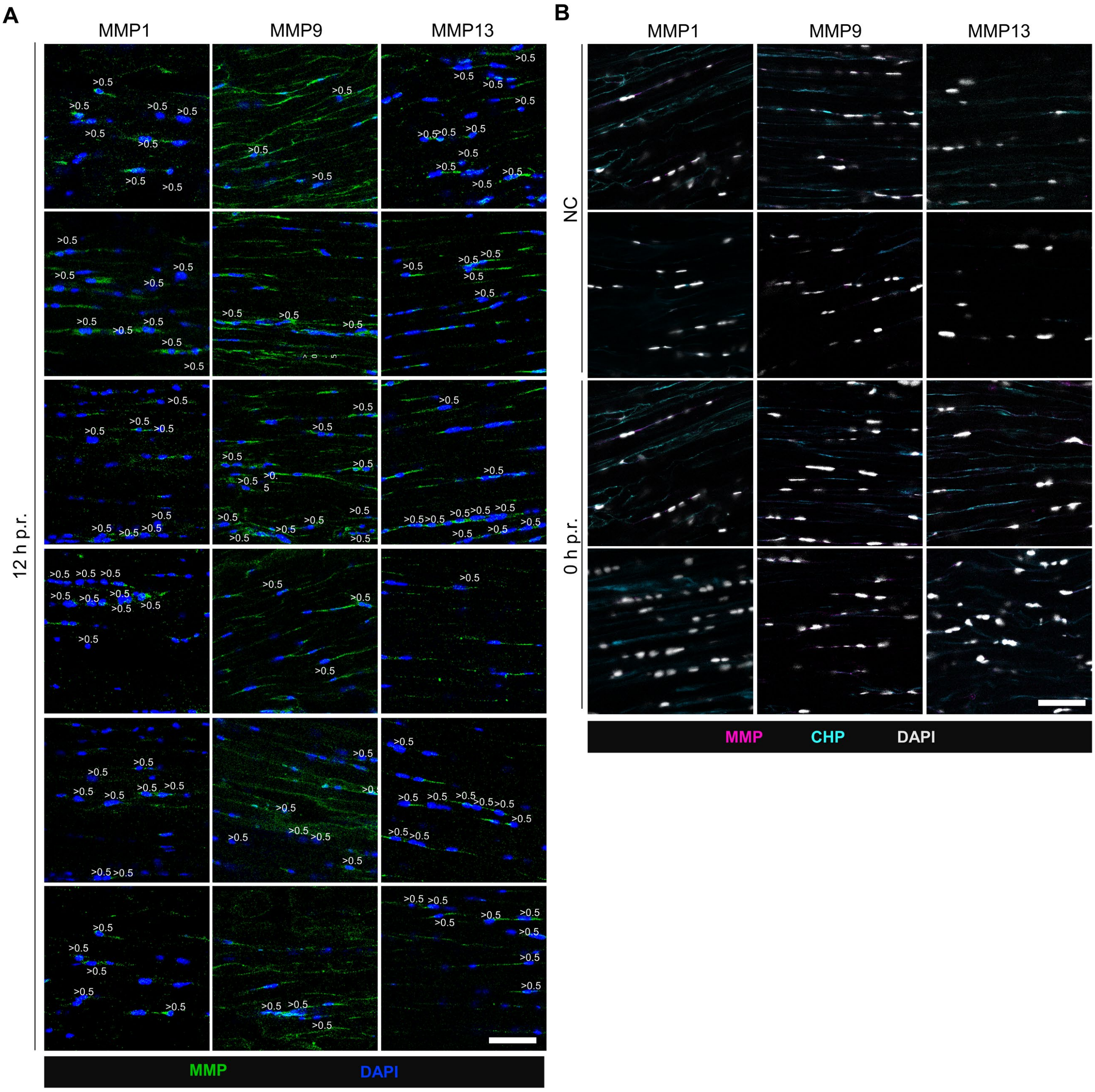

Fig. S10. DAPI and MMPs immunostaining of the rat Achilles tendons from the 12 h p.r. group. (A) Confocal fluorescence microscopy images of cryosections of the rat Achilles tendons from the 12 h p.r. group stained with DAPI (blue) and MMP antibodies (green) showed high populations of round tenocyte nuclei. The nuclei with a circularity greater than 0.5 were marked. Strong MMP immunofluorescence signals were primarily located near the tenocytes with high nuclear circularity. Six microscopic views randomly acquired from 6 slides were displayed for each MMP stain. **(B)** Representative confocal fluorescence microscopy images of the rat Achilles tendon cryosections from the 0 h p.r. and NC groups, stained with DAPI, Cy5-CHP, and anti-MMP antibodies. The results from the 12 h p.r. group are in Fig. 3C. The images represent similar results from at least five rats within each group. Scale bars: 50 μm.

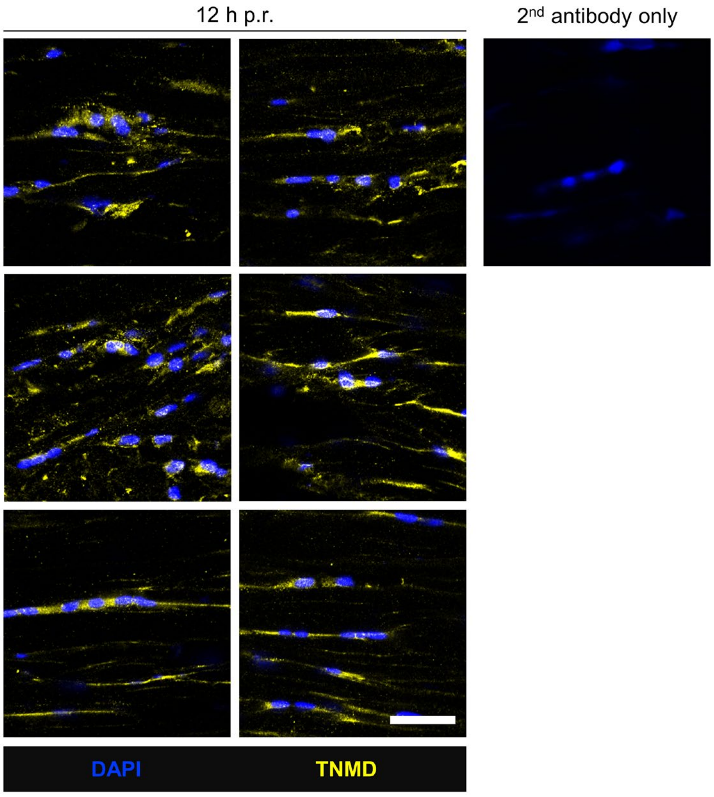

Fig. S11. Representative confocal fluorescence microscopy images of the rat Achilles tendon cryosections from the 12 h p.r. group, stained with DAPI and an anti-tenomodulin (TNMD) antibody. TNMD is a membrane protein marker specific for tenocytes. For the 2nd-antibody-only group, the tissue sections were stained as usual without using the primary anti-TNMD antibody (as a negative control). These images validated that the cells with the rounded nuclear morphology are indeed tenocytes and not immune or vascular cells. The images represent similar results from five rats within each group. Scale bar: 50 μm.

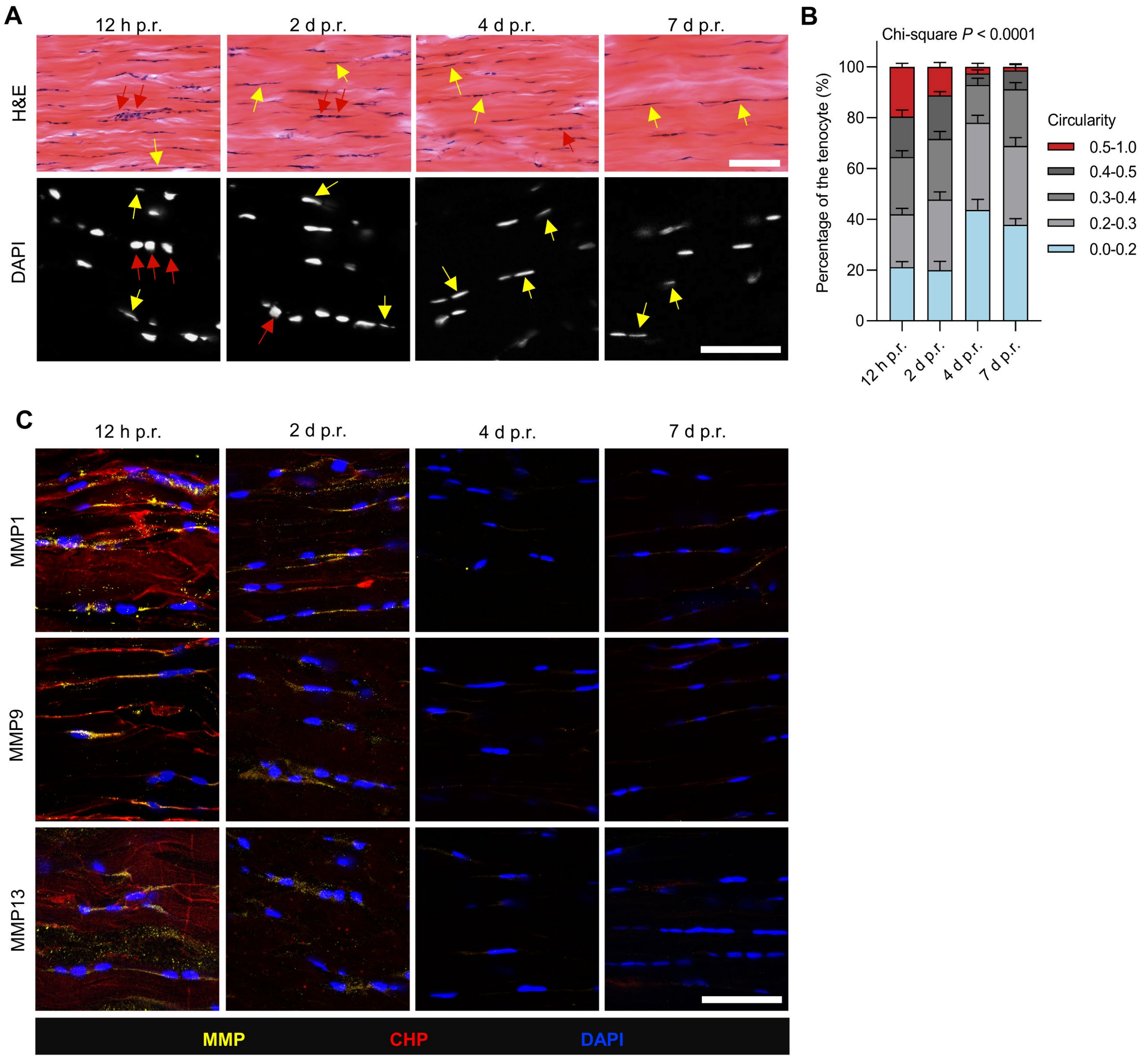

Fig. S12. Morphological changes and MMP expression in the Achilles tenocytes during the post-run recovery. **(A)** Micrographs showing the H&E and DAPI staining of the Achilles tendon cryosections from the recovery phase following 1-hour treadmill running (yellow arrows: elongated nuclei, red arrows: round nuclei). **(B)** The percentage of the tenocytes with various nuclei circularities quantified from the confocal microscopy images of the DAPI staining of 12 Achilles tendon sections from each group (3 fields of view per slide, data were expressed as mean + s.e.m., $P < 0.0001$, chi-squared test). **(C)** Representative confocal fluorescence microscopy images of the rat Achilles tendon cryosections from the recovery phase following 1-hour treadmill running, stained with DAPI, Cy5-CHP, and anti-MMP antibodies. The images represent similar results from four rats within each group **(A, C)**. Scale bars: 75 μm **(A, C)**.

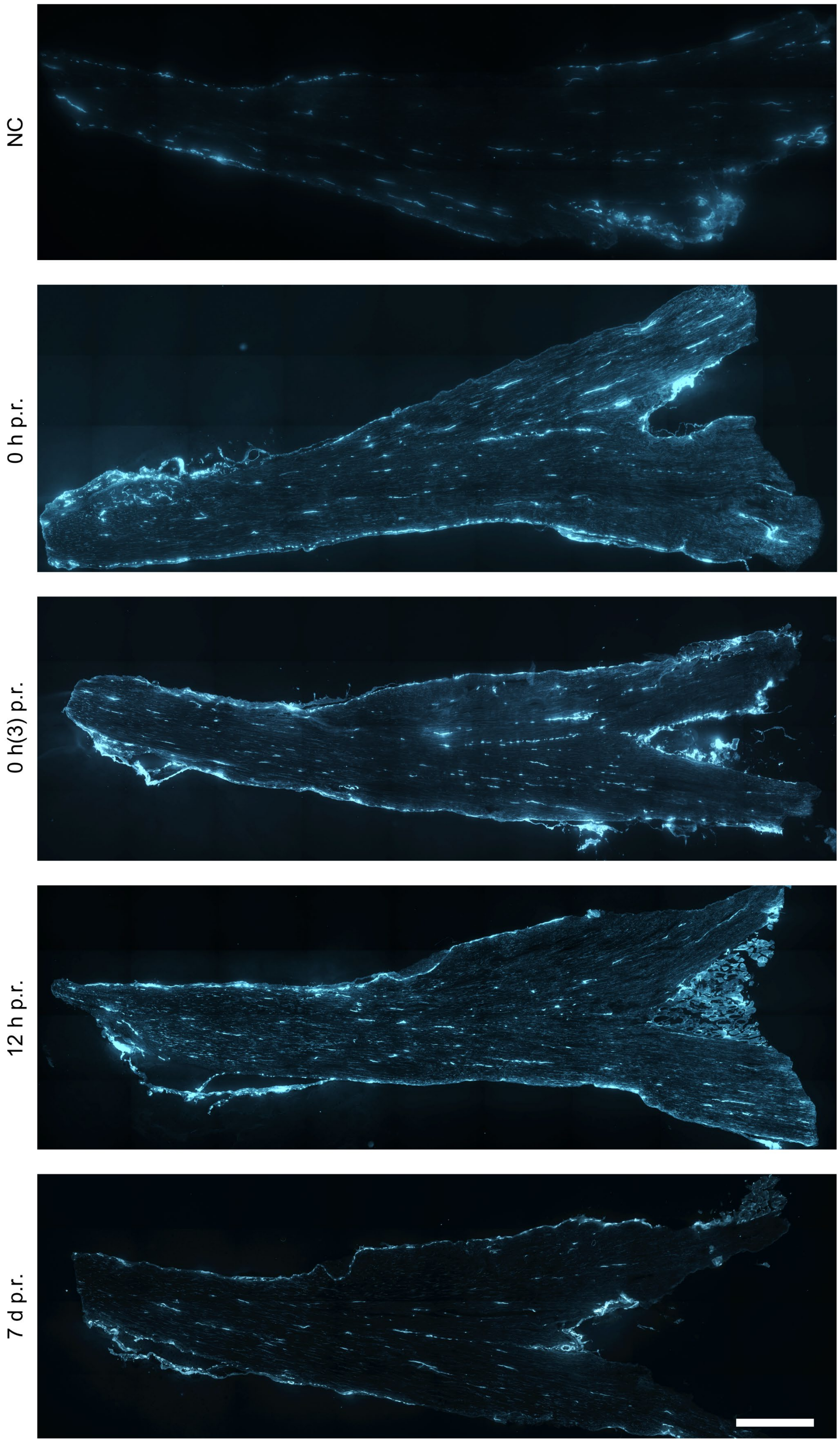

Fig. S13. The α-SMA immunostaining of the rat Achilles tendons from the one-time treadmill experiment. Fluorescence scans of α-SMA immunostaining of cryosections of the rat Achilles tendons from the one-time treadmill running experiment. Each image was representative of similar results from 6 rats within each group. Scale bar: 1 mm.

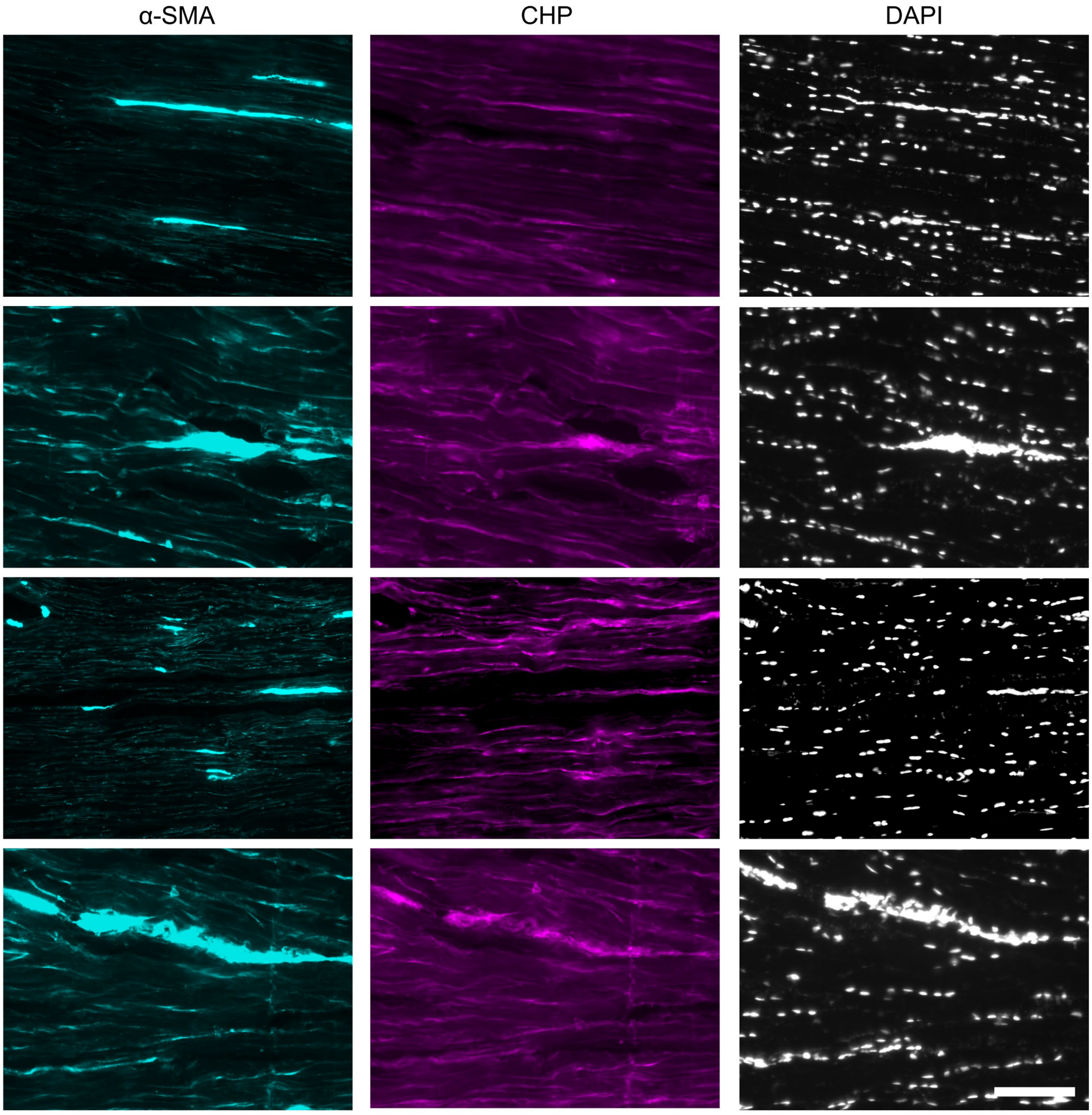

Fig. S14. The CHP, α-SMA, and DAPI co-staining of the rat Achilles tendons in the 12 h post-run group. Representative fluorescence images of the cryosections of SD rats' Achilles tendon in the 12 h post-run group stained with an α-SMA antibody (cyan), Cy5-CHP (magenta), and DAPI (white). The images revealed CHP fluorescence intensity in close proximity to the activated tenocytes with a high α-SMA expression at 12 h post-run. Representative microscopic views randomly acquired from 6 slides were displayed. Scale bar: 75 μm.

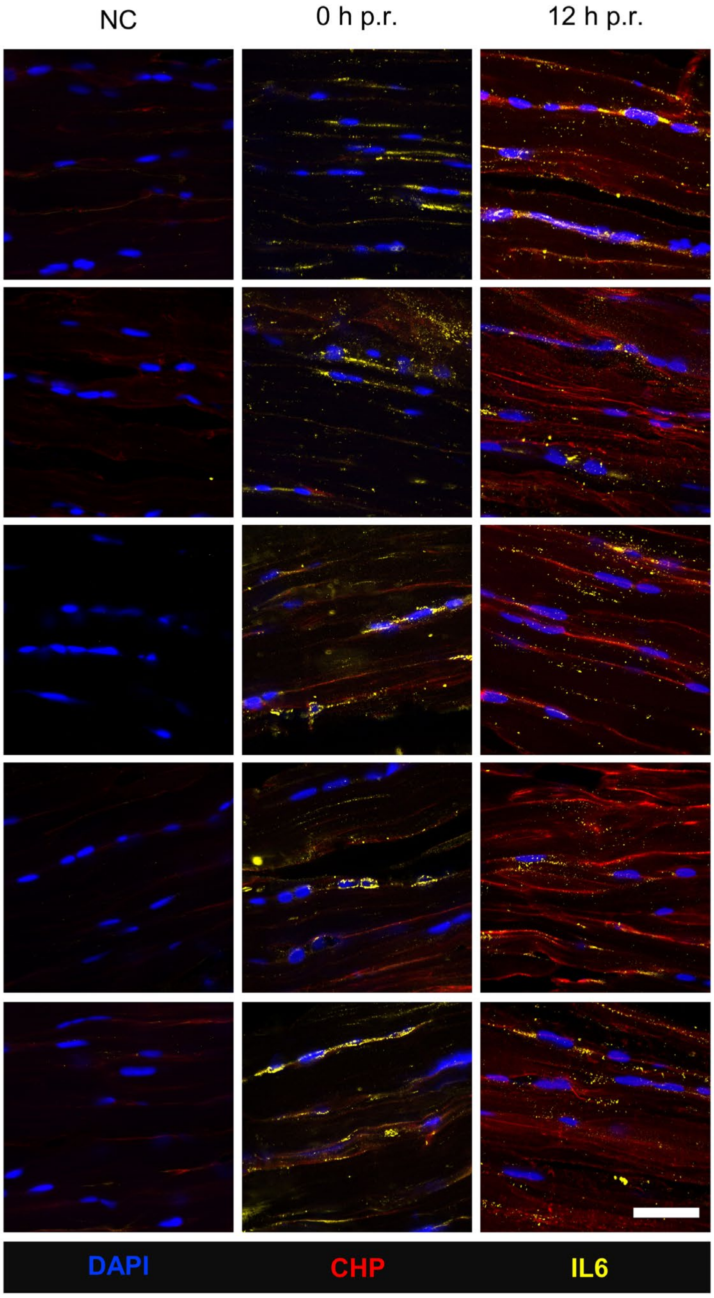

Fig. S15. Representative confocal microscopy images of the rat Achilles tendon cryosections collected at 12 h post-run, stained with DAPI, CHP, and an anti-IL6 antibody. These images indicated a notably up-regulated protein expression of IL6 at 0 h p.r. (particularly around the tenocyte nuclei), but to a reduced extent at 12 h p.r. The images represent similar results from five rats within each group. Scale bar: 50 μm.

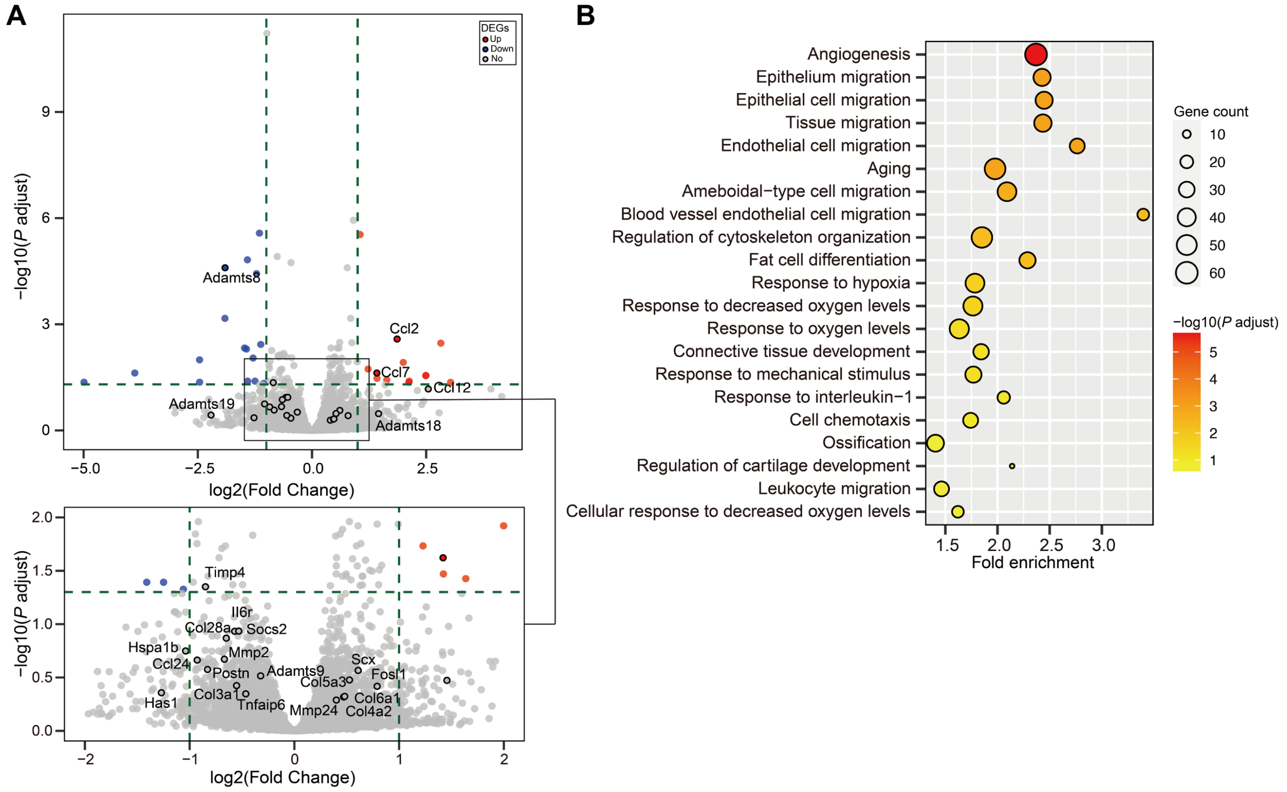

Fig. S16. RNA-seq analysis of the Achilles tendons from the NC and 12 h p.r. rats. (A) Volcano plot of the differentially expressed genes (12 h p.r. versus NC; fold change ≥ 2; adjust *P* value < 0.05). Significantly up-regulated genes are represented in red. Significantly down-regulated genes are in blue. Nonsignificant genes are in gray. Green vertical dotted lines highlight log fold changes of −1 and 1, whereas the green horizontal dotted line represents −log10(adjust *P* value) of 1.30. According to this threshold: we found up-regulation of the expression of 51 protein-coding genes and down-regulation of the expression of 42 protein-coding genes in the Achilles tendon at 12 h p.r. compared to the NC group. **(B)** GO enrichment analysis for the rat Achilles tendons at 12 h p.r. showing that the fold enrichment for the biological processes displayed in Fig. 4C, including responses to hypoxia or mechanical stimulus, was notably decreased.

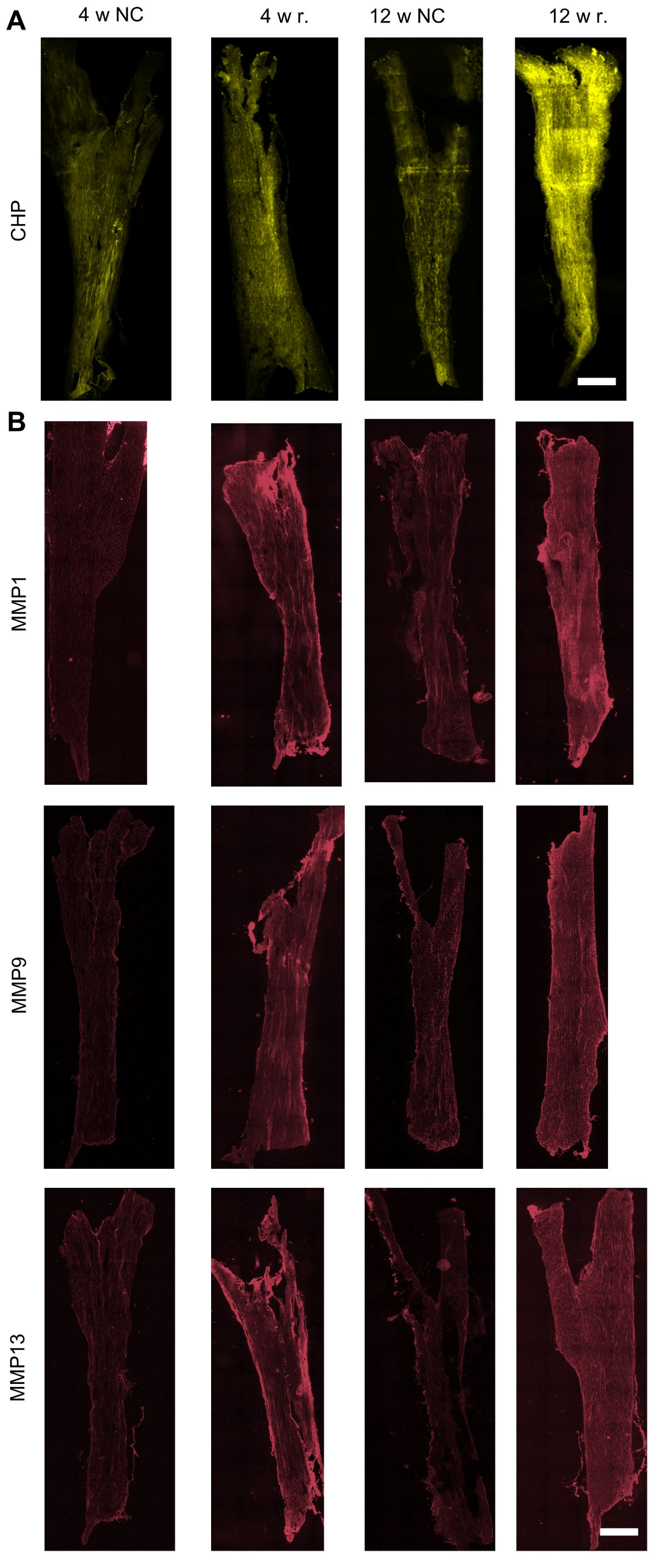

Fig. S17. The CHP and MMP immunostaining of the rat Achilles tendons from the long-term treadmill running experiment. Fluorescence scans of the Cy5-CHP staining **(A)** and MMP1, 9, 13 immunofluorescence staining **(B)** of cryosections of the Achilles tendons of the SD rats from the running groups (4 w r. and 12 w r.) and control group (4 w NC and 12 w NC). Each image was representative of similar results from 6 rats within each group. Scale bars: 1 mm.

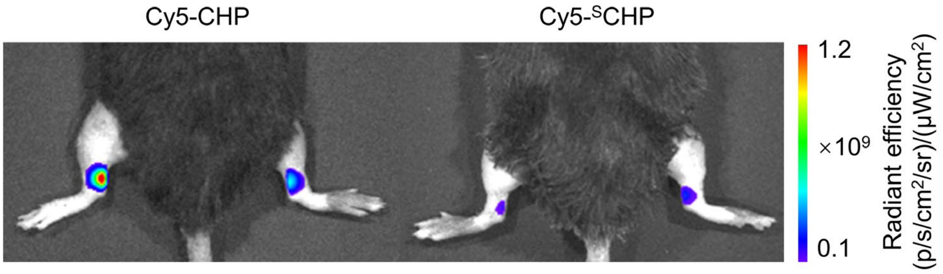

Fig. S18. *In vivo* fluorescence imaging of CHP hybridization in mice with collagenase-induced Achilles tendon injury. Representative *in vivo* fluorescence images of the mice with collagenase-injured Achilles tendons (left ankles) 2 h after subcutaneous, peritendinous injection of 0.04 nmol of Cy5-CHP / Cy5-^{S}CHP to each ankle. Denatured collagen was undetectable by our control probe Cy5-^{S}CHP with a scrambled CHP sequence (Cy5-Ahx-OfGGOfGfGfOfOGOfGOOfGGOOffG, MADLI-MS, calcd 3343.3 $[M+Na]^{+}$, observed 3343.6 $[M+Na]^{+}$). Each image was representative of similar results from three mice within each group.

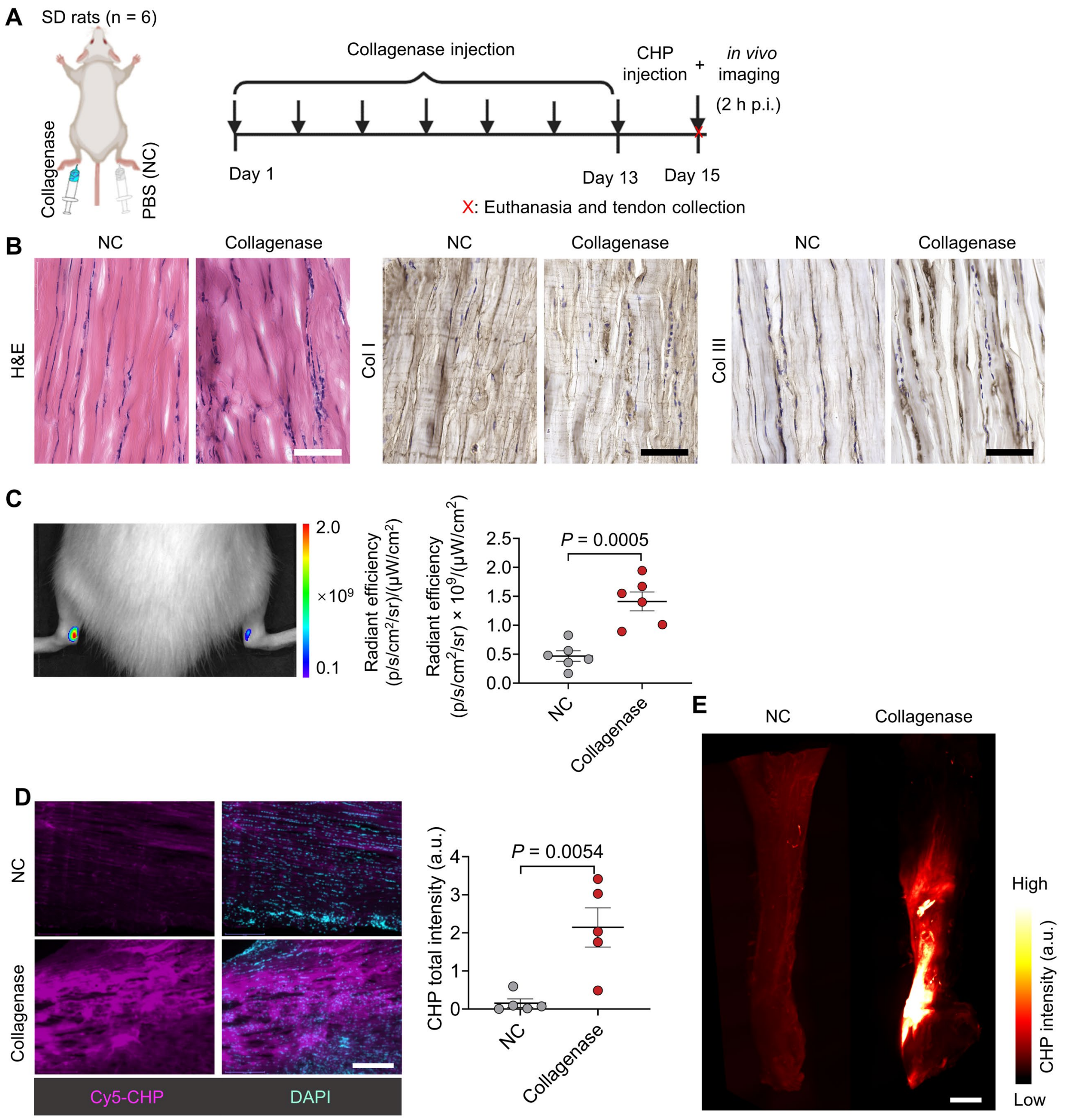

Fig. S19. ***In vivo*** **fluorescence imaging of collagen denaturation in a rat model of collagenase-induced Achilles tendon injury. (A)** Schematic of the SD rat model of the collagenase-injured Achilles tendon and the *in vivo* imaging timeline. Collagenase I (5 mg/mL in 60 µL of TESCA buffer solution) was injected subcutaneously in the left Achilles tendons of the rats every 2 days for 2 weeks to induce the Achilles tendon injury. Probe Cy5-CHP (0.1 nmol in 5 µL) was injected subcutaneously into both of the Achilles tendons of each rat 2 h before *in vivo* fluorescence imaging. **(B)** H&E staining and immunohistochemical staining showed disordered collagen fibers with slightly reduced collagen I and increased collagen III contents in the collagenase-injected rat Achilles tendons. **(C)** Representative *in vivo* fluorescence images and quantitative results of the rats with the collagenase-injected Achilles tendons showed that CHP fluorescence in the diseased ankles was about 2.8 times that of the control side. **(D)** Fluorescence images and quantitative analysis of cryosections showed that the CHP fluorescence intensity from the collagenase-injured tendons was over 10 times higher than that of the control ones. **(E)** Light-sheet fluorescence microscopy imaging of a model rat's Achilles tendons stained by Cy5-CHP *in vitro*. Each image was representative of similar results from all the rats within each group. Scale bars: 75 µm **(B&D)**, and 1500 µm **(E)**. Data were expressed as mean ± s.e.m.; statistical analysis: t-test **(C&D)**. The fig. S19A was created with BioRender.com.

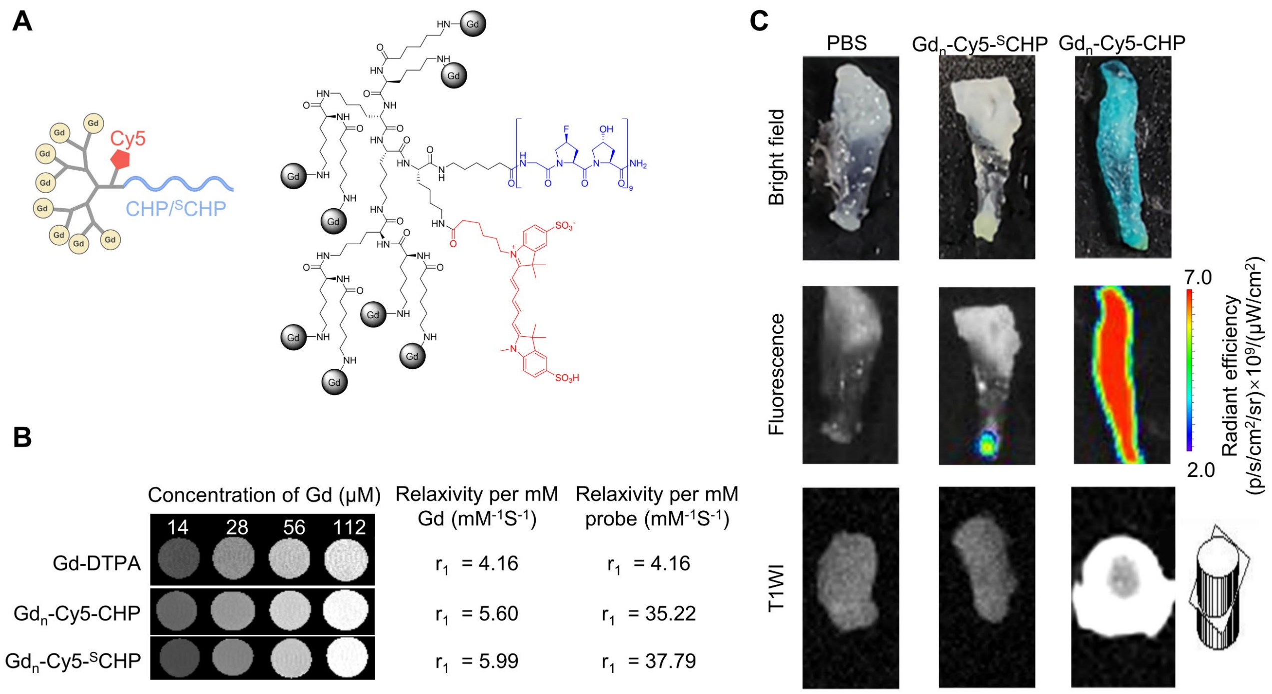

Fig. S20. Design and the *in vitro* T1-weighted MRI enhancement testing of the fluorescence MR contrast agent Gd_n-Cy5-CHP. (A) Schematic and chemical structures of the fluorescent MR probe Gd_n-Cy5-CHP. **(B)** T1 relaxivity of Magnevist (Gadopentetate Dimeglumine, Gd-DTPA), Gd_n-Cy5-CHP, and Gd_n-Cy5-^{S}CHP measured by 9.4 T magnetic resonance (MR) *in vitro*. **(C)** *In vitro* MR and fluorescence imaging of heat-denatured rat Achilles tendons treated with the Gd_n-Cy5-CHP probes. As positive controls to test the binding of the probes, nine rat Achilles tendon samples were heat denatured in boiling water for 1 min before being separated into three groups to be incubated at 4 °C overnight in 5 mL of PBS, Gdn-Cy5-CHP, and Gdn-Cy5-^{S}CHP, respectively (n = 3, probe concentration: 5 μM). Following extensive wash with PBS, the samples were fluorescently imaged with an IVIS Spectrum imager and scanned by a 9.4 T MRI scanner using a T1-RARE sequence. Under the bright field, the tendons incubated with Gd_n-Cy5-CHP were stained blue. The fluorescence intensity of the Achilles tendons stained with Gd_n-Cy5-CHP was extremely high. Strong signals were only visible from the group treated with Gd_n-Cy5-CHP in the transverse cross-sections of the samples scanned by MR using T1-RARE sequence. Each image was representative of similar results from three samples within each group.

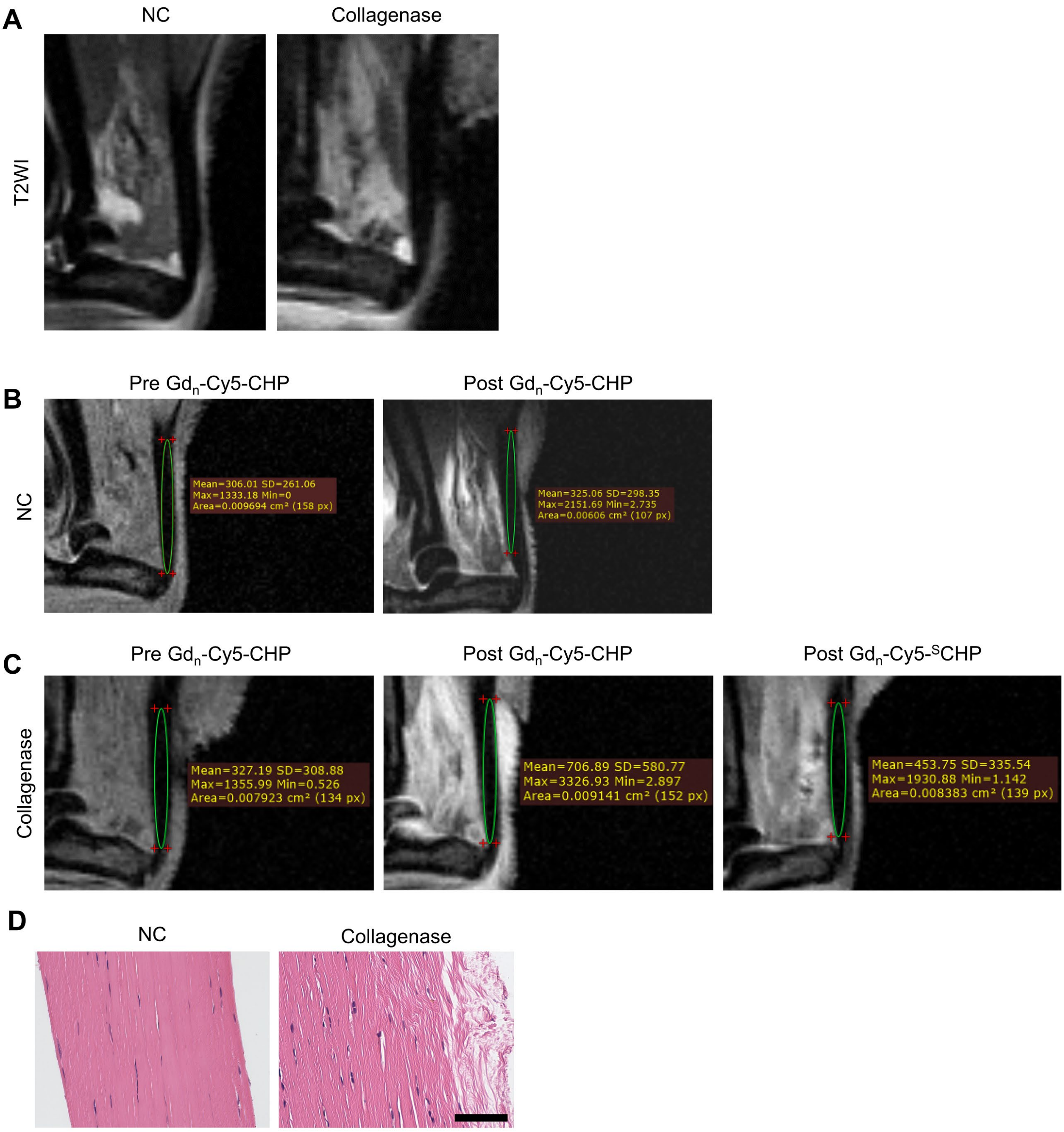

Fig. S21. Magnetic resonance imaging and histology of collagen-injured Achilles tendon in a mouse model. **(A)** Conventional sagittal scans with a T2W sequence of the model mice's normal and diseased Achilles tendons failed to show the lesion of Achilles tendinopathy. T2-FSE sequence: (TE = 30.0 ms; TR = 1500 ms; slice thickness: 0.3 mm; FOV = 20 × 20 mm; matrix dimensions = 228 × 228; bandwidth = 219.3 kHz; ETL = 8). **(B-C)** Illustration of the regions of interest (ROI) within the Achilles tendons for the quantitative analysis of the averaged T1WI signals pre- and post-injection of Gd_n-Cy5-CHP or Gd_n-Cy5-^{S}CHP. **(D)** The H&E histology images of the Achilles tendons collected from the model mice following *in vivo* MRI, which showed loose and irregularly aligned collagen fibers at the peripherals of the diseased tendons. Scale bar: 75 μm **(D)**.

Tables

Table S1. Design of the animal studies.

Model	Animal	Study aim	Group sizes	Schematic	Remark
One-time treadmill running	SD rats	To study molecular collagen damage following one-time running	Five groups (n = 6 per group):<br>- 0 h post-run<br>- 0 h(3) post-run<br>- 12 h post-run<br>- 7 d post-run<br>- normal control	Fig. 1D	
		To study molecular collagen denaturation during post-run resting	Four groups (n = 4 per group):<br>- 12 h post-run<br>- 2 d post-run<br>- 4 d post-run<br>- 7 d post-run	fig. S8A	
Long-term treadmill running	SD rats	To study molecular collagen damage following long-term repeated running	Four groups (n = 5 per group):<br>- 4-week running<br>- 12-week running<br>- 4-week control<br>- 12-week control	Fig. 5A	
Collagenase-injured tendons	SD rats	To study and fluorescently image the pathological, molecular collagen damage in the Achilles tendons in vivo in small animals	n = 6:<br>- Left ankle: PBS injection<br>- Right ankle: collagenase injection	fig. S19A	We carried out studies in both mice and rats to verify and exclude the possible species differences in rodents.
	C57BL/6 mice		n = 5:<br>- Left ankle: PBS injection<br>- Right ankle: collagenase injection	Fig. 6A	
	C57BL/6 mice	To image the pathological, molecular collagen damage by MR in the Achilles tendons in vivo in small animals.	n = 5:<br>- Left ankle: PBS injection<br>- Right ankle: collagenase injection	Fig. 7A	We chose to perform this study in mice because the collagenase-injury model is commonly utilized and reported in mice to mimic human tendinopathy. Also, it is cost-inhibitive to develop and test the Gd_n-Cy5-CHP agents in rats due to the drastically higher dosage compared to mice.

Table S2. Running distance of SD rats in the one-time treadmill running groups.

Group	Running time (h)	Running distance (m)
0 h p.r.	1	943.7 ± 154.6
0 h(3) p.r.	3	2752.6 ± 269.3
12 h p.r.	1	973.2 ± 119.1
2 d p.r.	1	1009.8 ± 17.9
4 d p.r.	1	1001.0 ± 19.0
7 d p.r.	1	964.2 ± 128.4

Table S3. Top 20 most significant biological processes differentially regulated in rat Achilles tendons at 0 h post-running from GO analysis. Differentially expressed genes (geneID) enriched in these biological processes are also included in the table.

Biological process description	p value	p.adj	q value	geneID	Count
response to lipopolysaccharide	3.66×10^{-15}	1.65×10^{-11}	1.15×10^{-11}	Tnfaip3/Ptgir/Zfp36/Aldoa/Tnfrsf26/Pde4d/Noct/S100a9/Hmgcs2/Ptger3/Il6/Il23r/Irak2/Timp4/Mgst1/Il1rn/Il1b/Lbp/Cebpb/Pck1/Pde4b/Zc3h12a/Cnr2/Alpl/Hif1a/Fos/Serpina1/Tnfrsf11b/Trib1/Csf2rb/Icam1/Arid5a/Fn1/Cxcl2/Cxcl1/Ppargc1a/Cd55/Ptgs2/Fmo1/Selp/Litaf/Socs1/Havcr2/Map2k3/Ccl2/Ccl5/Mpo/Csf3/Socs3/Gch1/Wnt5a/Jund/Hpgd/Cd86/Cldn1/Nod2/Junb/Bcr/Gja1/Serpine1/Nos1	61
response to molecule of bacterial origin	6.37×10^{-15}	1.65×10^{-11}	1.15×10^{-11}	Tnfaip3/Ptgir/Zfp36/Aldoa/Tnfrsf26/Pde4d/Noct/S100a9/Hmgcs2/Ptger3/Il6/Il23r/Irak2/Timp4/Mgst1/Il1rn/Il1b/Lbp/Cebpb/Pck1/Pde4b/Zc3h12a/Cnr2/Alpl/Hif1a/Fos/Serpina1/Tnfrsf11b/Trib1/Csf2rb/Icam1/Arid5a/Fn1/Cxcl2/Cxcl1/Ppargc1a/Cd55/Ptgs2/Fmo1/Selp/Litaf/Socs1/Havcr2/Map2k3/Ccl2/Ccl5/Mpo/Csf3/Socs3/Gch1/Wnt5a/Jund/Hpgd/Cd86/Cldn1/Nod2/Junb/Bcr/Gja1/Cd24/Serpine1/Nos1	62
response to hypoxia	2.76×10^{-14}	4.46×10^{-11}	3.11×10^{-11}	Hif3a/Aldoa/Pygm/Ccnb1/Ccna2/Mgarp/Postn/Rwdd3/Ddah1/Lep/Apold1/Nr4a2/Cat/Il1b/Bmp2/Angpt4/Pck1/Car9/Plk3/Fosl2/Kcnk3/Egln3/Hif1a/Slc8a3/Serpina1/Myc/Mmp13/Icam1/Ldlr/Epor/Pkm/Smad3/Mst1/Higd1a/Slc29a1/Nppc/Ppargc1a/Cd38/Adora1/Ptgs2/Crp/Stc2/Myocd/Ccl2/Socs3/Ajuba/Stc1/Ryr2/Prkcq/Suv39h2/Plat/Casr/Adipoq/Egr1/Hmox1/Cd24/Serpine1/Nos1	58
response to decreased oxygen levels	3.45×10^{-14}	4.46×10^{-11}	3.11×10^{-11}	Hif3a/Ppp1r15a/Aldoa/Pygm/Ccnb1/Ccna2/Mgarp/Postn/Rwdd3/Ddah1/Lep/Apold1/Nr4a2/Cat/Il1b/Bmp2/Angpt4/Pck1/Car9/Plk3/Fosl2/Kcnk3/Egln3/Hif1a/Slc8a3/Serpina1/Myc/Mmp13/Icam1/Ldlr/Epor/Pkm/Smad3/Mst1/Higd1a/Slc29a1/Nppc/Ppargc1a/Cd38/Adora1/Ptgs2/Crp/Stc2/Myocd/Ccl2/Socs3/Ajuba/Stc1/Ryr2/Prkcq/Suv39h2/Plat/Casr/Adipoq/Egr1/Hmox1/Cd24/Serpine1/Nos1	59
response to oxygen levels	7.42×10^{-14}	7.67×10^{-11}	5.35×10^{-11}	Hif3a/Ppp1r15a/Aldoa/Pygm/Ccnb1/Ccna2/Mgarp/Postn/Rwdd3/Ddah1/Lep/Apold1/Nr4a2/Cat/Il1b/Bmp2/Angpt4/Pck1/Car9/Plk3/Fosl2/Kcnk3/Egln3/Hif1a/Slc8a3/Serpina1/Txnrd1/Myc/Mmp13/Icam1/Ldlr/Epor/Pkm/Smad3/Mst1/Higd1a/Slc29a1/Nppc/Ppargc1a/Cd38/Adora1/Ptgs2/Crp/Stc2/Myocd/Ccl2/Socs3/Ajuba/Stc1/Ryr2/Prkcq/Suv39h2/Wnt5a/Plat/Casr/Adipoq/Egr1/Hmox1/Cdkn1a/Cd24/Serpine1/Nos1	62
response to acid chemical	1.60×10^{-12}	1.38×10^{-09}	9.59×10^{-10}	Gipr/Ffar2/Dgat2/Aldh1a1/Cyp26a1/Rbp4/Ccnb1/Bche/Glrb/Hmgcs2/Adh6/Il6/Lep/Mest/Cat/Il1b/Bmp2/Cebpb/Pck1/Gdap1/Klf4/Zc3h12a/Fabp3/Wnt4/Col4a6/Osr1/Hif1a/Serpina1/Myc/Col2a1/Icam1/Ldlr/Epor/Fn1/Ppargc1a/Cd38/Bcl11a/Myog/Ptgs2/Rxrg/Socs1/Grin2a/Dusp1/Trim16/Ccl2/Ccl5/Krt13/Sox9/Myh6/Sncg/Nat1/Acsl1/Plat/Adamts1/Runx1/Hes1/Cldn1/Adipoq/Egr1/Cdo1/Gstt1/Gja1	62
fat cell differentiation	1.12×10^{-11}	8.26×10^{-09}	5.76×10^{-09}	Zfp36/Clip3/Ffar2/Cebpa/Pex11a/Fam57b/Lgals12/Ffar4/Fabp4/Noct/Lep/Nr4a2/Frzb/Bmp2/Cebpb/Runx1t1/Nr4a3/Klf4/Zc3h12a/Wif1/Nr4a1/Smad3/Ppargc1a/Ptgs2/Socs1/Sox8/Msx2/Id4/Wnt5a/Mrap/Hes1/Adipoq/Cebpd/Snai2/Egr2/Retn/Medag/Trpv4	38
blood circulation	1.61×10^{-11}	1.04×10^{-08}	7.26×10^{-09}	Dll1/Ppp1r13l/Csrp3/Sult1a1/Pcsk5/F2rl1/Pde4d/Npr3/Postn/Casq2/Ddah1/Ptger3/Lep/Adra2b/Edn3/Hrh3/Pde4b/Zc3h12a/Kcne5/Hif1a/Icam1/Scn2b/Smad3/Tpm1/Htr1b/Cxcr2/Nppc/Alb/Corin/Cd38/Adora1/Ptgs2/Cacna1e/Crp/Adra1b/Map2k3/Alox12/Uts2r/Gch1/Myh6/Adra1a/Stc1/Ryr2/Prkcq/Casr/Adipoq/Dsg2/Hbegf/Hmox1/Irx5/Mylk3/Agt/Ier3/Bcr/Gja1/Nos1/Trpv4	57
circulatory system process	2.28×10^{-11}	1.31×10^{-08}	9.12×10^{-09}	Dll1/Ppp1r13l/Csrp3/Sult1a1/Pcsk5/F2rl1/Pde4d/Npr3/Postn/Casq2/Ddah1/Ptger3/Lep/Adra2b/Edn3/Hrh3/Pde4b/Zc3h12a/Kcne5/Hif1a/Icam1/Scn2b/Smad3/Tpm1/Htr1b/Cxcr2/Nppc/Alb/Corin/Cd38/Adora1/Ptgs2/Cacna1e/Crp/Adra1b/Map2k3/Alox12/Uts2r/Gch1/Myh6/Adra1a/Stc1/Ryr2/Prkcq/Casr/Adipoq/Dsg2/Hbegf/Hmox1/Irx5/Mylk3/Agt/Ier3/Bcr/Gja1/Nos1/Trpv4	57
response to purine-containing compound	4.61×10^{-11}	2.38×10^{-08}	1.66×10^{-08}	Ezr/Fosb/Ppp1r15a/Aqp8/Fosl1/Pygm/Pde4d/Hmgcs2/Casq2/Il6/Mat2a/Il1b/Pck1/Fosl2/Hif1a/Slc8a3/Fos/Cib2/Ptprn/Ppargc1a/Ptgs2/Dusp1/Ccl2/Sphk1/Stc1/Crem/Ryr2/Jund/Plat/Atp7b/Adipoq/P2rx6/Egr1/Cdo1/Junb/Serpine1	36
ossification	6.08×10^{-11}	2.86×10^{-08}	1.99×10^{-08}	Cebpa/Acan/Ifitm5/Noct/Bglap/Lep/Atp6v0a4/Cat/Grem1/Bmp2/Cebpb/Impad1/Runx3/Wnt4/Alpl/Phex/Six2/Osr1/Hif1a/Col2a1/Sp7/Nfe2/Mmp13/Smad3/Ccr1/Fn1/Ihh/Nppc/Spp1/Mepe/Ibsp/Dmp1/Fignl1/Myog/Ptgs2/Sox8/Fgf18/Sost/Sox9/Fasn/Stc1/Msx2/Id4/Wnt5a/Jund/Runx1/Casr/Cebpd/Snai2/Junb/Egr2/Gja1	52
connective tissue development	1.05×10^{-10}	4.53×10^{-08}	3.16×10^{-08}	Gpr4/Acan/Dgat2/Sox6/Hmgcs2/Wnt2b/Lep/Slc25a25/Frzb/Cd44/Grem1/Thbs1/Bmp2/Impad1/Runx3/Timp1/Six2/Osr1/Hif1a/Col2a1/Mmp13/Barx2/Smad3/Arid5a/Ihh/Nppc/Ppargc1a/Sox8/Fgf18/Sox9/Stc1/Msx2/Id4/Wnt5a/Runx1/Casr/Snai2/Egr1/Trpv4	39
response to mechanical stimulus	1.64×10^{-10}	6.54×10^{-08}	4.56×10^{-08}	Fosb/Csrp3/Acan/Bag3/Fosl1/Ccnb1/Slc1a3/Postn/Bglap/Il6/Thbs1/Il1b/Bmp2/Slc2a1/Nrxn1/Fosl2/Hif1a/Fos/Myc/Col2a1/Mmp13/Stra6/Ihh/Serpine2/Kit/Btg2/Chi3l1/Ptgs2/Ccl2/Mpo/Sost/Sox9/Ryr2/Jund/Egr1/Junb/Agt/Hspa1b/Gja1/Retn/Serpine1/Nos1	42

cellular divalent inorganic cation homeostasis	5.01×10^{-10}	1.73×10^{-07}	1.20×10^{-07}	Gipr/Gpr4/Csrp3/Trpm1/Cemip/Pygm/F2rl1/Pde4d/Rxfp3/Casq2/Ptger3/Aplnr/Il1b/Jph2/Edn3/Hrh3/Slc24a2/Pgm1/Gpr3/Gpr157/Atp2b3/Kcnk3/Slc8a3/Gpr65/Epor/Cib2/Smad3/Ccr1/Cxcr2/Cxcl2/Cxcl1/Cd38/Cd55/Slc41a1/Grin2a/Stc2/Adra1b/Ccl2/Ccl5/Uts2r/Adra1a/Stc1/Slc39a14/Lpar6/Ryr2/Mt1/Atp7b/Casr/Mt2A/Agt/Gja1/Cd24/Nos1/Trpv4	54
positive regulation of cell migration	7.41×10^{-10}	2.40×10^{-07}	1.67×10^{-07}	Wdr62/Cemip/Pcsk5/Kif20b/F2rl1/Pde4d/Postn/Ptger3/Tcaf2/Atoh8/Tnfaip6/Thbs1/Sema6d/Il1b/Bmp2/Angpt4/Lbp/Edn3/Zc3h12a/Hif1a/Myc/Icam1/Plet1/Smad3/Ccr1/Ptp4a1/Fn1/Cxcr2/Ackr3/Cxcl2/Cxcl1/Kit/Ptgs2/Tnfsf18/Sele/Selp/Fgf18/Alox12/Ccl2/Ccl7/Ccl5/Itga3/Sox9/Sphk1/Wnt5a/Irs2/Snai2/Egr1/Hbegf/Foxf1/Agt/Retn/Serpine1/Trpv4	54
response to organophosphorus	9.87×10^{-10}	2.91×10^{-07}	2.03×10^{-07}	Ezr/Fosb/Aqp8/Fosl1/Pygm/Pde4d/Hmgcs2/Mat2a/Il1b/Pck1/Fosl2/Slc8a3/Fos/Cib2/Ptprn/Ptgs2/Dusp1/Trim16/Ccl2/Sphk1/Stc1/Crem/Jund/Plat/Atp7b/Adipoq/P2rx6/Egr1/Cdo1/Junb/Serpine1	31
divalent inorganic cation homeostasis	1.05×10^{-09}	2.91×10^{-07}	2.03×10^{-07}	Gipr/Gpr4/Csrp3/Trpm1/Cemip/Pygm/F2rl1/Pde4d/Rxfp3/Casq2/Ptger3/Aplnr/Il1b/Jph2/Edn3/Hrh3/Slc24a2/Pgm1/Gpr3/Gpr157/Atp2b3/Kcnk3/Slc8a3/Gpr65/Epor/Cib2/Smad3/Ccr1/Cnnm4/Cxcr2/Cxcl2/Cxcl1/Cd38/Cd55/Slc41a1/Grin2a/Stc2/Adra1b/Ccl2/Ccl5/Uts2r/Adra1a/Stc1/Slc39a14/Lpar6/Ryr2/Mt1/Atp7b/Casr/Mt2A/Agt/Gja1/Cd24/Nos1/Trpv4	55
multi-multicellular organism process	1.07×10^{-09}	2.91×10^{-07}	2.03×10^{-07}	Fosb/Fosl1/Pcsk5/Ptger3/Lep/Il1rn/Acvr1c/Adra2b/Slc2a1/Wnt4/Timp1/Fosl2/Hif1a/Fos/Slc38a1/Mmp13/Epor/Mst1/Ihh/Serpine2/Corin/Cd38/Lif/Cd55/Ptgs2/Stc2/Havcr2/Ccl2/Itga3/Sphk1/Stc1/Hpgd/Aqp4/Dsg2/Junb/Agt/Gja1/Serpine1/Nos1	39
regulation of blood circulation	1.18×10^{-09}	3.05×10^{-07}	2.13×10^{-07}	Csrp3/F2rl1/Pde4d/Casq2/Ptger3/Lep/Adra2b/Edn3/Pde4b/Zc3h12a/Kcne5/Hif1a/Icam1/Scn2b/Tpm1/Cd38/Adora1/Ptgs2/Cacna1e/Adra1b/Gjd3/Uts2r/Gch1/Myh6/Adra1a/Stc1/Ryr2/Prkcq/Casr/Dsg2/Hbegf/Irx5/Agt/Gja1/Nos1	35
muscle cell proliferation	2.95×10^{-09}	7.26×10^{-07}	5.06×10^{-07}	Tnfaip3/Ptgir/Tenm4/Pcsk5/Rbp4/Ccnb1/Pde4d/Npr3/Il6/Thbs1/Nr4a3/Klf4/Hif1a/Trib1/Myc/Tpm1/Htr1b/Nppc/Ppargc1a/Myog/Ptgs2/Myocd/Alox12/Ccl5/Hpgd/Adipoq/Egr1/Hbegf/Hmox1/Agt/Pim1/Gja1/Retn	33

Table S4. Top 20 most significant biological processes differentially regulated in rat Achilles tendons at 12 h post-running from GO analysis. Differentially expressed genes (geneID) enriched in these biological processes are also included in the table.

Biological process description	p value	p.adj	q value	geneID	Count
angiogenesis	3.79×10^{-10}	1.99×10^{-6}	1.76×10^{-6}	Rspo3/Pdcd6/Gpr4/Rasip1/Rnh1/Thbs4/Esm1/Pik3ca/Sfrp2/Rbm15/F3/Emcn/C1galt1/Cav1/Epha1/Vhl/Plxnd1/Apold1/Egfl7/Abl1/Eng/Calcrl/Aplnr/Angpt4/Pofut1/Sox18/B4galt1/Klf4/Tie1/Ephb2/Foxo4/Vash1/Notch3/Angptl4/Elk3/Hdac7/Nr4a1/Bmper/Ets1/Ctsh/Hyal1/Ccl2/Ccl12/Tmem100/Hoxb3/Hdac5/Sphk1/Loxl2/Egr3/Rgcc/Jcad/Angpt2/Col4a2/Fgf1/Cx3cl1/Mmp2/Cdh13/Pnpla6/Ephb4/Ccl24	60
tissue migration	4.54×10^{-7}	0.000922	0.000814	Pdcd6/Atoh8/Actg2/Vhl/Plxnd1/Strap/Abl1/Angpt4/Sox18/Klf4/Vash1/Akt1/Ptprr/Hdac7/Nr4a1/Amotl1/Bmper/Ets1/Ctsh/Hyal1/Tgfbr3/Tgfb2/Prox1/Pdpk1/Pfn1/Itga3/Hdac5/Loxl2/Egr3/Rgcc/Jcad/Angpt2/Hbegf/Fgf1/Cdh13/Ephb4/Scarb1	37
epithelial cell migration	5.74×10^{-7}	0.000922	0.000814	Pdcd6/Atoh8/Vhl/Plxnd1/Strap/Abl1/Angpt4/Sox18/Klf4/Vash1/Akt1/Ptprr/Hdac7/Nr4a1/Amotl1/Bmper/Ets1/Ctsh/Hyal1/Tgfbr3/Tgfb2/Prox1/Pdpk1/Pfn1/Itga3/Hdac5/Loxl2/Egr3/Rgcc/Jcad/Angpt2/Hbegf/Fgf1/Cdh13/Ephb4/Scarb1	36
epithelium migration	7.02×10^{-7}	0.000922	0.000814	Pdcd6/Atoh8/Vhl/Plxnd1/Strap/Abl1/Angpt4/Sox18/Klf4/Vash1/Akt1/Ptprr/Hdac7/Nr4a1/Amotl1/Bmper/Ets1/Ctsh/Hyal1/Tgfbr3/Tgfb2/Prox1/Pdpk1/Pfn1/Itga3/Hdac5/Loxl2/Egr3/Rgcc/Jcad/Angpt2/Hbegf/Fgf1/Cdh13/Ephb4/Scarb1	36
aging	1.26×10^{-6}	0.001204	0.001064	Nup62/Ctsc/Picalm/Ucp3/Arntl/Ypel3/Smc5/Mme/Ngf/Vcam1/F3/Cisd2/Cav1/Rxra/Abl1/Eng/Bcl2l1/Pck1/C1qb/Foxo4/Xdh/Eif2s1/Tgfb3/Vash1/Akt1/Eef2/Nuak1/Socs2/Hspa8/Ncam1/Eef1a1/Gsta1/Col3a1/Creb1/Ppargc1a/Igfbp3/Tgfb2/Kcnmb1/Ccl2/Ppp1r9b/Aoc3/Top2b/Myh6/Htr2a/Hnrnpk/Npy1r/Adrb3/Apod/Cldn1/Cx3cl1/Amfr/Mmp2/Hmga1/Jmjd1c	54
endothelial cell migration	1.37×10^{-6}	0.001204	0.001064	Pdcd6/Atoh8/Vhl/Plxnd1/Abl1/Angpt4/Sox18/Klf4/Vash1/Akt1/Hdac7/Nr4a1/Amotl1/Bmper/Ets1/Prox1/Pdpk1/Hdac5/Loxl2/Egr3/Rgcc/Jcad/Angpt2/Fgf1/Cdh13/Ephb4/Scarb1	27
ameboidal-type cell migration	2.82×10^{-6}	0.002116	0.001869	Pdcd6/Has1/Lrp5/Sema3c/Atoh8/Vhl/Plxnd1/Strap/Abl1/Acvr1c/Angpt4/Sdc4/Sox18/Klf4/Vash1/Akt1/Ptprr/Hdac7/Nr4a1/Amotl1/Bmper/Ets1/Ctsh/Hyal1/Sema3f/Tgfbr3/Tgfb2/Prox1/Pdpk1/Pfn1/Itga3/Hdac5/Loxl2/Egr3/Rgcc/Jcad/Angpt2/Arhgef7/Hbegf/Fgf1/Cdh13/Gna12/Ephb4/Scarb1	44
blood vessel endothelial cell migration	7.51×10^{-6}	0.004933	0.004358	Vhl/Abl1/Angpt4/Sox18/Klf4/Vash1/Akt1/Hdac7/Nr4a1/Amotl1/Hdac5/Egr3/Rgcc/Jcad/Angpt2/Ephb4/Scarb1	17
fat cell differentiation	1.02×10^{-5}	0.00579	0.005115	Sirt2/LOC108348122/Atf5/Tph1/Arntl/Lrp5/Wwtr1/Sfrp2/Steap4/Rarres2/Wnt5b/Adig/Nr4a3/Klf4/Akt1/Wif1/Nr4a1/Zfp385a/Zbtb16/Smad6/Creb1/Per2/Lrrc8c/Ppargc1a/Lamb3/Nr1d1/Htr2a/Id4/Ccdc3/Adrb3/Hes1/Zfpm1	32
regulation of cytoskeleton organization	1.10×10^{-5}	0.00579	0.005115	Myadm/Cdc42ep5/LOC108348122/Atf5/Nup62/Akap13/Mapk3/Cttn/Rbm14/Map3k1/Stmn2/Cav1/LOC100909784/Epha1/Arhgef5/Dctn1/Abl1/Sdc4/Tmem67/Lpar1/Asap3/Mid1/Plekhh2/Tgfb3/Mdm1/Triobp/Pick1/Parp3/Tpr/Tgfb2/Prox1/Arf1/Arhgap44/Pfn1/Ssh2/Inpp5k/Myo1c/Spag5/Cltc/Stmn4/Rgcc/Mk1/Dlc1/Cep97/Diaph1/Synpo/Chmp1a/Hspa1b/Ranbp2/Arpc1b/Fscn1/Ccl24/Hip1r	53
endothelial cell proliferation	1.36×10^{-5}	0.006518	0.005758	Pdcd6/Thbs4/F3/Cav1/Atoh8/Egfl7/Eng/Xdh/Vash1/Akt1/Nr4a1/Bmper/Prox1/Ccl2/Loxl2/Egr3/Rgcc/Jcad/Cdh13/Ccl24/Scarb1	21
iron ion transport	2.00×10^{-5}	0.008753	0.007732	LOC100360087/Picalm/Steap4/Steap2/B2m/Ftl1/Heph/LOC100359668/Rab11b/LOC100362384/Cltc/Slc39a14	12
hexose metabolic process	2.18×10^{-5}	0.008798	0.007772	Akt2/LOC499235/Fuom/Cpt1a/Lrp5/Bad/Pik3ca/Pofut1/Pck1/B4galt1/LOC108351137/Wdtc1/Eno1/G6pd/Akt1/Cry1/Per2/Ppargc1a/Man2b2/Mrfap1/Igfbp3/Atf3/Inpp5k/Pdk2/Nr1d1/Npy1r/Apod/Ptpn2/Ranbp2/Hk1	30
regulation of epithelial cell migration	2.61×10^{-5}	0.008987	0.007939	Pdcd6/Atoh8/Strap/Abl1/Angpt4/Klf4/Vash1/Akt1/Ptprr/Hdac7/Amotl1/Bmper/Ets1/Ctsh/Hyal1/Tgfbr3/Tgfb2/Prox1/Pdpk1/Pfn1/Itga3/Hdac5/Rgcc/Jcad/Angpt2/Hbegf/Fgf1	27
positive regulation of fat cell differentiation	2.64×10^{-5}	0.008987	0.007939	Tph1/Lrp5/Sfrp2/Rarres2/Wnt5b/Adig/Akt1/Wif1/Zfp385a/Zbtb16/Creb1/Htr2a/Ccdc3	13
regulation of vesicle-mediated transport	2.77 × 10-^{5}	0.008987	0.007939	Prkcg/Ehd2/Axl/Akt2/LOC108348122/Nr1h2/Tbc1d17/Mex3b/Picalm/Arrb1/Mapk3/Tbc1d12/Cav1/B2m/Tbc1d20/Bcl2l1/Sdc4/Tbc1d2/Atp13a2/Gpc3/Vsnl1/Rab11b/Scyl2/Pick1/Rab3d/Hspa8/Ncam1/Rab8b/Evi5/Fcer1a/Pdpk1/Arf1/Dlg4/Ccl2/Unc13d/Tbc1d16/Lgi3/Gas1/Hnrnpk/Lman2/Stam/Syt15/Adprhl1/Tnk2/Rit2/Tbc1d9/Cdh13/Btbd9/Gopc/Hip1/Scarb1/Hip1r/Atp2a2	53

regulation of actin filament-based process	2.91 × 10^{-5}	0.008987	0.007939	Myadm/Cdc42ep5/Akap13/Cttn/Map3k1/Sri/Cav1/LOC100909784/Epha1/Arhgef5/Abl1/Sdc4/Lpar1/Pde4b/Asap3/Plekhh2/Frmd6/Tgfb3/Triobp/Pick1/Hcn4/Tnnt2/Tgfb2/Prox1/Arf1/Arhgap44/Pfn1/Ssh2/Inpp5k/Myo1c/Jup/Rgcc/Mk1/Dlc1/Dsc2/Synpo/Arpc1b/Fscn1/Ccl24/Hip1r/Atp2a2	41
positive regulation of cell migration	3.22 × 10^{-5}	0.009098	0.008037	Pdcd6/Myadm/Akt2/Mapk3/Thbs4/Postn/F3/Sema3c/Epha1/Rarres2/Atoh8/Wnt5b/Abl1/Tnfaip6/Angpt4/Lpar1/Foxo4/Akt1/Hdac7/Amotl1/Ets1/Ctsh/Hyal1/Tnfsf18/Selp/Tgfb2/Prox1/Pfn1/Ssh2/Myo1c/Ccl2/Ccl7/Ccl12/Itga3/Mien1/Aoc3/Sphk1/Jcad/Plvap/Arhgef7/Hbegf/Diaph1/Fgf1/Cd74/Cx3cl1/Mmp2/Ripor1/Cdh13/Fbxo31/Ccar1/Ccl24	51
activation of immune response	3.42 × 10^{-5}	0.009098	0.008037	Lilrb3a/Nop53/C5ar2/Nectin2/Kcnn4/Rbm14/Cav1/Skap2/Abl1/Tyro3/Nr4a3/Pde4b/Sfpq/Gpatch3/C1qb/C1qc/Tlr7/Tspan6/Nfkbia/Cactin/Irak3/Xrcc6/Cacnb3/Plscr1/Cd38/Cd55/C4bpa/Susd4/Tlr5/Pdpk1/Lcp2/Irf1/Tnip1/Nr1d1/Sec14l1/Rgcc/Cblb/Ptpn2/Hspa1b	39
vesicle organization	3.46 × 10^{-5}	0.009098	0.008037	LOC100910446/Pdcd6/Bloc1s3/Nectin2/Akt2/Plekhf1/Tbc1d17/Picalm/Sytl2/LOC100361543/Stx3/Tbc1d12/Hps1/Fnbp1l/Tmem127/Tbc1d20/Sdc4/Vapb/Tbc1d2/Stx12/Plekhj1/Laptm4b/Zfp385a/Rfx2/Creb1/Ptprn/Evi5/Arf1/Dlg4/Rilp/Tbc1d16/Stam/Syt15/Adprhl1/Snx2/Tbc1d9/Stx2	37

Table S5. Running protocol used for the long-term treadmill running groups.

		Duration (min)	Speed (m/min)
Week 1	Day 1	20	10-15
	Day 2	30	10-15
	Day 3	Rest	-
	Day 4	40	15-17
	Day 5	50	15-17
	Day 6	60	15-17
	Day 7	Rest	-
Week 2 - 12	Run on days 1, 2, 4, 5, and 6 each week, rest on days 3 and 7	60	20

Hyphenated values in the speed column represent the gradual increase in speed at the beginning of each running session. Running began at the lower speed and was increased by 1 m/min each minute until the higher speed was achieved. Rats ran at this higher speed for the remainder of the duration.

Table S6. Primer sequences in this study.

Gene	Accession number		Sequences	Product length (bp)
Mmp1	NM_001134530	Forward:	5' TGGATGACTCTCACTGTGGTC 3'	123
		Reverse:	5' AGCAATGTGTTTCCTCTTCGT 3'	
Mmp9	NM_031055	Forward:	5' CCCATGTATCACTACCACGAGG 3'	91
		Reverse:	5' GGTCAGGTTTAGAGCCACGAC 3'	
Mmp13	NM_133530	Forward:	5' AAGACTGTGCGAACTGGACAG 3'	89
		Reverse:	5' CACTAAGGAAAGCAGGGAAGG 3'	
Gapdh	NM_017008.4	Forward:	5' ACGGGAAACCCATCACCATC 3'	80
		Reverse:	5' CACGACATACTCAGCACCAGCA 3'	

MMP: matrix metallopeptidase; GAPDH: glyceraldehyde-3-phosphate dehydrogenase.

Other Supplementary Materials:

MOVIES

Movie S1. Video of rats running on a treadmill in a typical session.

Movie S2. Light sheet fluorescence microscopy imaging showing Cy5-CHP's *in vitro* binding to denatured collagen molecules within a cleared Achilles tendon, harvested from a rat model of collagenase-induced Achilles tendon injury, stained with Cy5-CHP overnight.

Movie S3. Light sheet fluorescence microscopy imaging showing Gd_n-Cy5-CHP's *in vivo* binding to denatured collagen molecules within a cleared Achilles tendon from a mouse model of collagenase-induced Achilles tendon injury. Probe Gd_n-Cy5-CHP was subcutaneously injected into the peri-Achilles tendon tissues 6 h before *in vivo* MR imaging, after which the samples were collected.

Data S1. Original data for Figs. 1-3 and Figs. 5-7 in tabular format.
